# Wildfire‐Driven Changes in Terrestrial Subsidies Shift Freshwater Microbial and Zooplankton Communities to New Compositional States

**DOI:** 10.1111/mec.17794

**Published:** 2025-05-20

**Authors:** Margaret Y. Demmel, Christopher B. Wall, Cody J. Spiegel, Natalia Erazo, Evelyn M. Diaz, Madeline G. Perreault, Elisabet Perez‐Coronel, Sara L. Jackrel, Jeff S. Bowman, Jonathan B. Shurin

**Affiliations:** ^1^ Division of Biological Sciences, Department of Ecology, Behavior and Evolution University of California San Diego California USA; ^2^ Department of Earth Sciences University of Hawai‘i at Mānoa Honolulu Hawaii USA; ^3^ Scripps Institution of Oceanography University of California San Diego California USA

**Keywords:** browning, freshwater microbiomes, terrestrial subsidies, wildfire, zooplankton

## Abstract

Wildfire frequency and intensity are increasing globally, impacting terrestrial and aquatic ecosystems. Deposition of burned materials into aquatic environments can affect biotic communities and nutrient cycling. We investigated how post‐fire terrestrial deposition shapes microbial and zooplankton community composition and function across time by manipulating plant material amount (loading; 0–400 g) and chemical composition (burned vs. unburned) in 400 L experimental mesocosms over four months. Burning treatment had minimal effects (1.4%), while loading (6.6%) and time (19.2%) contributed significantly to free‐living microbial community variation. Dramatic changes in environmental conditions and microbiome composition occurred at a 50–100 g loading threshold within 30 days. High‐loading mesocosms showed hypoxia, increased dissolved organic carbon and aromaticity, elevated bacterial density, and shifts in bacterial community function relating to enhanced carbon degradation, suggesting efficient microbial use of carbon resources despite low oxygen and increased water colour. Zooplankton communities were primarily influenced by time (24.9%), with loading (10.3%) and burning (2.3%) having weaker effects. Zooplankton community composition shifted at a 100 g–150 g threshold that persisted over time, with crustaceans declining and mosquito larvae dominating at higher loading levels. Zooplankton‐ and plant detritus‐associated microbiomes were distinct but showed minimal treatment effects after four months, indicating greater environmental filtering for these microhabitats relative to horizontal transmission from treatment‐altered water microbiomes. In contrast, free‐living microbiomes maintained loading‐driven compositional differences, while predicted genome traits and functions converged across treatments. These results suggest that post‐wildfire deposition drives zooplankton and microbial communities into distinct compositional states punctuated by abrupt transitions, but microbiomes may recover community‐level functionality over time.

## 1 Introduction

Climate change and human activity are increasing the frequency and severity of wildfires (Pausas and Keeley 2021; Jones et al. 2022). Fire is a cross‐ecosystem disturbance that affects nutrient dynamics within terrestrial (soil and plant) and aquatic ecosystems by increasing deposition of burned materials into waterways (Bixby et al. 2015; Rhoades et al. 2019; Butler et al. 2021). Pyrogenic materials can alter aquatic production and basal resources supplied to food webs (Cooper et al. 2015) and the abundance of aquatic taxa (i.e., fishes, macroinvertebrates) (Cooper et al. 2015; Whitney et al. 2015). Thus, fire‐driven changes to both the chemical composition and volume of allochthonous inputs to aquatic systems may shape water chemistry, macro‐ and microorganism communities, and ecosystem processes including system metabolism, greenhouse gas fluxes, and nutrient cycling (Bixby et al. 2015; Blanchet et al. 2022).

Fire affects the quantity (loading amount) and quality of allochthonous organic material in freshwater habitats (Paul et al. 2022), and each can have distinct impacts on communities and ecosystem function. Terrestrial loading increases dissolved organic carbon (DOC) and nutrient availability for both autotrophic and heterotrophic microorganisms, promoting net primary productivity at low‐loading levels (Thrane et al. 2014; Solomon et al. 2015), but inducing light limitation (due to increased aromaticity and water colour) and hypoxia or anoxia as microbial respiration decreases dissolved oxygen (DO) (Blanchet et al. 2022; Wall et al. 2024; Spiegel et al. 2024). Fire chemically transforms the stoichiometry of terrestrial material (Butler et al. 2020), which may amplify or dampen loading effects in aquatic ecosystems by altering dissolved and particulate material pools (Paul et al. 2022; Rhoades et al. 2011). For instance, burning can decrease the availability of microbially accessible (labile) carbon from plant material (Thuile Bistarelli et al. 2021), reducing trophic transfer of allochthonous nutrients to zooplankton consumers (Wall et al. 2024) and increasing contributions of primary production to food webs (Cooper et al. 2015). Moderate fire effects may therefore benefit zooplankton by increasing short‐term consumption of nutritional phytoplankton as opposed to terrestrial material (Brett et al. 2009). Alternatively, more extreme or long‐term fire‐induced chemical transformations of plant material can also alter resource quality, which may increase the abundance of aquatic microorganisms that degrade refractory compounds (Thuile Bistarelli et al. 2021; Seo et al. 2009) or organisms (zooplankton, macroinvertebrates, fishes) that can tolerate the biogeochemical conditions produced by pyrogenic materials in waterways (Cooper et al. 2015; Verkaik et al. 2015).

Post‐fire terrestrial deposition in aquatic ecosystems often occurs in a single‐pulsed disturbance where burning is followed by precipitation and run‐off; thereafter, ecosystems recover to previous stable states or transition to novel states (Dahm et al. 2015). However, different components of ecosystems may follow distinct trajectories. For example, debris flows from extreme fire events can lead to extirpation of fishes (Rieman et al. 2012), shifts in macroinvertebrate emergence and community composition (Malison and Baxter 2010), and increased turbidity and nutrient concentrations that persist for years (Rhoades et al. 2011). Following top‐down pulse‐disturbances, total community biomass of microorganisms and invertebrates (e.g., bacterial, phytoplankton, zooplankton) has been shown to rapidly return to pre‐disturbance levels, with similar functional roles in recovered communities despite changes in community composition (Urrutia‐Cordero et al. 2021; Hillebrand and Kunze 2020). However, these insights come primarily from observational studies (Whitney et al. 2015; Butler et al. 2020; Rhoades et al. 2011; Dahm et al. 2015; Malison and Baxter 2010), which, while invaluable for capturing natural complexity, are often limited in their ability to detect causal mechanisms of wildfire‐driven change and test for thresholds in ecosystem responses to fire. Furthermore, the majority of studies focus on physical or chemical ecosystem responses, with some identifying changes in vertebrates and macroinvertebrate communities (Rieman et al. 2012; Malison and Baxter 2010). Yet, fire impacts on microbial and plankton communities vital to aquatic ecosystem services and functions have largely been unexplored. Our experimental approach complements previous observational studies, allowing us to disentangle potential mechanisms of fire effects on aquatic ecosystems and identify ecological tipping points that may be obscured in observational studies.

We investigated the loading‐dependent effects of fire on terrestrial inputs and their influence on aquatic communities and ecosystem processes. We manipulated terrestrial material loading and its chemical composition by burning to test the consequences of wildfires on microbial and zooplankton communities (including both aquatic insect larvae and planktonic crustacean species) in experimental mesocosms through time. Specifically, we characterised bacterial community composition (using 16S‐rRNA gene sequencing) (i) found in water, (ii) associated with burned and unburned plant detritus, and (iii) associated with zooplankton crustaceans [*Daphnia* spp.] and planktonic stages (larvae/pupae) of mosquitoes [*Culex* spp.]. We also assessed zooplankton community composition, abundance of water‐associated autotrophic and heterotrophic microbes (via flow cytometry), and the carbon isotopic values of dissolved gases related to microbial metabolism and function (carbon dioxide (δ^13^CO_2_) and methane (δ^13^CH_4_)). Previous observational work has described unimodal relationships between DOC and primary productivity due to trade‐offs between nutrient and light limitation (Holgerson et al. 2022). We hypothesised that community composition, microbial cell densities, and carbon cycling would show similar nonlinear responses to plant loading, with cell densities peaking and communities diverging at an intermediate loading threshold. We hypothesised that these responses would differ between burned and unburned treatments, with potentially less extreme ecosystem responses to loading in burned relative to unburned treatments due to the deposition of primarily recalcitrant carbon (Brett et al. 2009). We further predicted that mesocosms would follow unique post‐disturbance recovery trajectories in the short term that would result in prolonged (~90d) differences in zooplankton/microbial community composition. Our goal was to determine whether functional or structural components of pond microbial communities show thresholds or critical transitions in response to loading of allochthonous organic matter, and whether these are altered by fire or change over time. Given increases in fire activity worldwide (Higuera et al. 2021; Liu et al. 2010), characterising zooplankton and microbial community dynamics following a fire‐driven disturbance and, in particular, identifying disturbance thresholds at which ecosystem change is observed, can improve our understanding of when and how aquatic systems may be at risk of destabilisation or biodiversity loss (McCullough et al. 2019).

## 2 Materials and Methods

### 2.1 Experimental Design

Thirty 400 L experimental mesocosms were used to test burning and loading effects on aquatic ecosystems, using a regression design to examine non‐linear relationships between plant loading and ecosystem response. Detailed methods were previously described (Wall et al. 2024; Spiegel et al. 2024). Briefly, mesocosms (burned or unburned, *n* = 15 per treatment) were filled with municipal water (28 October 2021), and stocked with an assemblage of live zooplankton (> 63 μm) collected from vertical tows at Lake Miramar, San Diego, CA. Mesocosms received stepwise additions of dried plant material (0 to 400 g total) from two shrubs abundant in western North America: *Salvia leucophylla* (Greene) (hereafter, sage) and *Salix lasiolepis* (Benth.) (hereafter, willow). Sage and willow leaves and stems (< 2 cm diameter) were dried (45°C) and either unburned or burned with a butane torch (see *Supporting Information*). On 5 November 2021, mesocosms received equal masses of sage and willow for their respective burning treatment in separate nylon bags (25 × 15 cm, 250 μm mesh). Two control mesocosms received zooplankton stock but no plant materials. A constant water level was maintained with weekly water additions from an adjacent 400 L reservoir.

### 2.2 Sampling Design and Response Metrics

**FIGURE 1:**
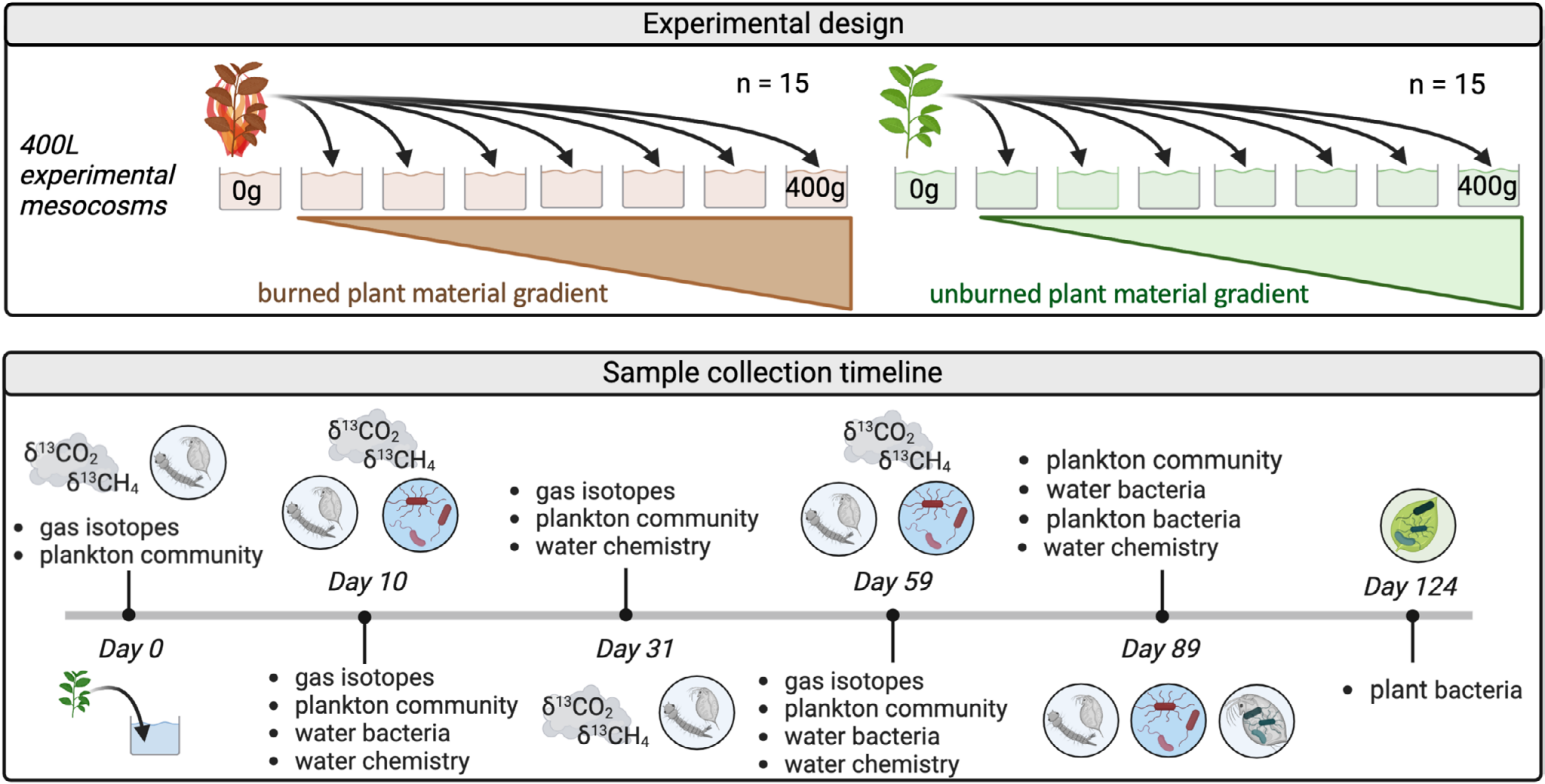
Schematic representation of experimental design and timeline of sample collection. Samples were processed as described in *Methods*. Plankton community refers to planktonic crustaceans and immature macroinvertebrates (mosquito larvae). Water chemistry refers to measurements of dissolved organic carbon, dissolved oxygen, and UV‐visible absorbance of dissolved organic matter.

Mesocosms were sampled six times, once before plant materials were added (3 November 2021, Day‐0) and five times after: Day‐10 (15 November), Day‐31 (6 December), Day‐59 (3 January), Day‐89 (2 February), and Day‐124 (8 March) (Figure 1). We sampled the zooplankton community five times (Days‐0, 10, 31, 59, 89), δ^13^CO_2_ and δ^13^CH_4_ four times (Days‐0, 10, 31, 59), free‐living water microbial communities (16S‐sequencing, flow cytometry) three times (Days‐10, 59, 89), and microbiomes (16S‐sequencing) of *Daphnia* zooplankton and mosquito larvae/pupae (hereafter, referred collectively as ‘zooplankton’) (Day‐89) and in‐tank plant materials once (Day‐124). Sampling the microbiome of zooplankton/larvae and plant detritus is necessarily destructive, as it requires removing a large quantity of zooplankton or plant detritus from the experimental mesocosms to acquire sufficient material for DNA extraction and 16S‐rRNA sequencing. Therefore, these samples could only be collected once to avoid disturbing the experiment at intermediate time points, so we only measured these metrics (zooplankton‐ and plant‐associated microbiomes) at the final sampling time point.

Environmental conditions and dissolved organic matter (DOM) chemistry were measured through Day 89, previously reported in (Wall et al. 2024; Spiegel et al. 2024) (see *Supporting Information* S1). To test the influence of environmental conditions and chemistry on microbial community composition at each time point, we used the following: percent dissolved oxygen (%DO), humification index (HIX, the degree of DOM humification), freshness index (BIX, indicating freshly produced DOM), fluorescence index (FI, the proportion of DOM originating from microbial vs. terrestrial sources), SUVA_254_ (a proxy for aromaticity), Sr. (indicating molecular weight of DOC), pH, and temperature (see Spiegel et al. 2024) for further detail on measurement methods).

### 2.3 Zooplankton Community Composition

We sampled zooplankton communities using an integrated 1 L vertical water sampler to collect water (~4 L) from each mesocosm. Zooplankton were filtered (> 63 μm) and preserved (70% ethanol), with densities and community composition determined using light microscopy and a counting wheel. Zooplankton were identified to the lowest taxonomic level and pooled as members of suborder Copepoda (calanoids (Calanoida), cyclopoids (Cyclopoida), nauplii), family Daphniidae (*Daphnia*, *Ceriodaphnia*, *Bosmina*), phyla Rotifera (*Kellicottia*, *Asplanchna*, *Keratella*), and Insecta (chironomids (Chironomidae), mayflies (Ephemeroptera)). Due to high abundance of larvae/pupae, mosquitoes (*Culex* spp.) were counted and represented separately from other macroinvertebrates.

### 2.4 Carbon Dioxide and Methane Carbon Isotope Analysis

Details for sampling of greenhouse gases (CO_2_, CH_4_) can be found in (Wall et al. 2024). Briefly, 25 mL of surface water (0.1 m depth) and 25 mL of ambient air were collected in a sterile 60 mL syringe, shaken (2 min) to reach equilibrium, and injected into evacuated Exetainers for gas quantification. Background traces of CO_2_ and CH_4_ on each sampling day were determined by collecting 12 mL of ambient air in evacuated Exetainers. Samples were stored upside down at room temperature and analysed at the University of California Davis Stable Isotope Facility within three weeks of collection (see Supporting Information).

### 2.5 Flow Cytometry

Flow cytometry samples were processed following (Wilson et al. 2022). Briefly, samples were prefiltered (60 μm), aliquoted into 2 mL tubes, and fixed to 0.25% glutaraldehyde. We ran samples on a Guava easyCyte 11HT (Luminex, Austin, TX, USA) using blue (488 nm) and violet (405 nm) lasers to evaluate the fluorescence signals of SYBR Green I (SG‐stained; ThermoFisher Scientific, Waltham, MA, USA) to estimate autofluorescent cell abundance and unstained chlorophyll‐containing samples to estimate absolute cell counts respectively (see Supporting Information).

### 2.6 Microbial Community Sampling

Unfiltered water samples (250 mL) were collected from the surface of each mesocosm in acid‐washed (10% HCl) Nalgene bottles, transported on ice to the Scripps Institution of Oceanography, and refrigerated (4°C). Water samples were immediately filtered (0.2 μm Supor membrane, Pall Corporation, New York, NY, USA) and stored (−80°C) until DNA extraction. *Daphnia* and mosquito larvae were collected (Day‐89) from mesocosms using a plankton tow and filter cups (> 63 μm), preserved (70% ethanol, −20°C), and sorted by microscopy into DNA extraction tubes (*Daphnia*: *n* = 50 individuals/tube, except one where *n* = 7; mosquito larvae: *n* = 15 individuals/tube, except one where *n* = 9) and stored (−80°C) until DNA extraction. *Daphnia* and/or mosquito larvae were found in high enough abundance for microbiome analysis in 22 of the 30 mesocosms. Plant detritus microbiomes in each mesocosm were sampled using decomposed plant material (1 g, removed on Day‐124) collected from each litter bag (for both sage and willow) with sterile forceps, placed in microcentrifuge tubes, and stored (−80°C) until DNA extraction.

### 2.7 DNA Extraction and 16S‐rRNA Gene Sequencing

DNA was extracted from all samples using the KingFisher Flex Purification System and MagMax Microbiome Ultra Nucleic Acid Extraction kit (ThermoFisher Scientific, USA) and quantified using the Qubit High Sensitivity DNA quantification kit. Extracted DNA was shipped to Argonne National Laboratory (ANL) for library preparation and 151 × 151 bp paired‐end Illumina MiSeq sequencing of the V4 region of the 16S‐rRNA gene with modified primers 515F‐806R (Walters et al. 2016) (see Supporting Information for further information).

### 2.8 Bioinformatics

Illumina Miseq reads were demultiplexed, and reads were quality controlled and denoised using the ‘FilterandTrim’ and ‘dada’ commands and assembled with the ‘mergePairs’ command in dada2 (Callahan et al. 2016). Merged amplicon sequence variants (ASVs) were directed into the paprica pipeline (Bowman and Ducklow 2015), which places ASVs on a reference tree constructed of full‐length 16S‐rRNA genes from all completed genomes in GenBank (Haft et al. 2018) using EPA‐ng (Barbera et al. 2019), Infernal (Nawrocki and Eddy 2013), and Gappa (Czech et al. 2020) for the phylogenetic placement. Unique reads were assigned to internal or terminal branches on the reference tree, and these placements were quantified as ‘edges’. We removed bacteria unidentified at the Phylum level, mitochondrial sequences, and chloroplast sequences based on reference tree taxonomies. Sequences assigned to phylum Cyanobacteria were additionally blasted to the ‘nr’ database to ensure they were not plant or algal chloroplast sequences. All filtered samples were rarefied to the minimum per‐sample sequencing depth (5258 reads). Statistical analyses of water, zooplankton, and plant detritus microbial community structure were carried out at the level of ASV, while taxonomic classification, genomic characteristic estimates, and metabolic predictions were made for edges. We quantified characteristics of water‐associated bacterial genomes by assigning edges to complete or partial microbial genomes within the paprica pipeline (Bowman and Ducklow 2015). We estimated metabolic pathways, average bacterial genome size (number of basepairs), number of coding sequences, bacterial growth rate, and relative bacterial genomic plasticity (Erazo and Bowman 2021). Growth rate was estimated based on an existing database of bacterial doubling times (Weissman et al. 2021). Relative genomic plasticity was calculated by determining, for a given bacterial strain, (a) the dissimilarity between the given 16S‐rRNA gene and all other identified bacterial genomes within the dataset; (b) the dissimilarity between the given bacterial proteome and all identified proteomes; then calculating the mean difference between (a) and (b). A larger difference between (a) and (b) for a given strain indicates that the functional difference (the proteome) between two strains is not accurately predicting the phylogenetic difference (the genome) between two strains, suggesting high genomic plasticity of this strain relative to other strains within the dataset. As relative genomic plasticity increases, a microbial genome has diverged further from the average genome of its clade (Bowman and Ducklow 2015). Functional annotations and genomic estimates are limited by incomplete databases for 16S‐sequences; many environmental microbes remain uncultured, thus functionally characterising all ASVs precisely remains impossible. (Lloyd et al. 2018). However, paprica has been used previously to identify metabolic pathways that benefit plant growth (Syiemiong and Rabha 2024; Raj et al. 2019) and denitrification (Erazo and Bowman 2021; Arfken et al. 2017) as well as to quantify community‐wide genomic characteristics (Wilson et al. 2022; Davila Aleman et al. 2024).

### 2.9 Statistical Analysis

All analyses were performed in R version 4.3.1 (R Core Team 2024). We tested the effects of treatment (burned and unburned), plant loading, and time (to account for potential pseudo‐replication in effects over time) on microbial communities in the water using permutational multivariate analysis of variance (PERMANOVA) tests with 999 permutations on a Bray–Curtis dissimilarity matrix using ‘vegan’ (Oksanen et al. 2022). Non‐metric multidimensional scaling (NMDS) was used to visualise loading effects in 50 g bins through time. We determined the loading threshold that explained the greatest microbial variation at Day‐10 by running multiple PERMANOVAs with loading amount (classified as less‐than‐or‐equal‐to all possible loading values (0–400 g)) as a single binary explanatory variable. The loading amount associated with the highest PERMANOVA *R* ^2^ value (amount of variation explained) was identified as the primary threshold of microbial community change. To test correlations between microbial composition and measured environmental variables or mean bacterial genome characteristics, we used *envfit* in ‘vegan’ for NMDS ordinations of microbial variation at each time point. To characterise the relationships between loading, environmental variation, and microbiome variation, we ran linear models and piecewise linear models using ‘segmented’ (Fasola et al. 2018). We characterised environmental variation as the first axis (PC1) of a principal coordinate analysis (PCoA) using nine environmental variables (%DO, [DOC], FI, BIX, HIX, Sr., SUVA_254_, pH, and temperature), and characterised microbiome variation as the first axis (NMDS1) of a NMDS plot showing microbiome composition. We then ran a linear model and a piecewise linear model (with a single unknown breakpoint identified by the model) for loading versus PC1 (environment) and PC1 vs. NMDS1 (microbiome), respectively. We compared the two models using an ANOVA to determine whether a linear or piecewise‐linear model best fit the data. The piecewise‐linear model was considered the better fit when *p* < 0.01.

We visualised microbial variation across sample types (water, zooplankton, plant detritus) at the final time point using PCoA and Bray–Curtis dissimilarity and calculated effects of treatment, plant mass, and sample type using PERMANOVA. To quantify similarity between different sample types across loading levels, we calculated the pairwise Bray–Curtis dissimilarity between all samples, subset to only samples occurring at the same loading levels, then used linear models or generalised additive models (GAMs; ‘mgcv’ [Wood 2011]) to determine variation in microbial similarity across loading levels.

Metabolic pathways in water microbial communities that differed significantly across plant loading levels were identified using ‘DESeq2’ (Love et al. 2014) at Day‐10 and Day‐89. Plant mass loading was centered and scaled to use as a continuous variable in the DESeq2 model. We identified the number of bacterial ‘edges’ (see *Bioinformatics* above) predicted to contain each pathway, and subset the final identification of differentially abundant pathways (*p* < 0.01; abs(log2fold) > 2) to only those that appeared in at least five bacterial taxa to ensure that identified pathways represented a potential community‐wide change, rather than gain or loss of a single microbe.

Treatment effects on zooplankton community, autofluorescence and total microbial cell abundance, greenhouse gas δ^13^C, and inferred genomic characteristics were analysed at each time point using generalised additive models (Wood 2011). Within each time point, we applied a model selection framework that allowed us to evaluate non‐linear and/or treatment‐specific offsets of response variables across the gradient of plant biomass loading and burning treatments (Pedersen et al. 2019) (see Supporting Information for further detail on model selection).

## 3 Results

### 3.1 Water, Zooplankton, and Plant Detritus Microbial Communities

**FIGURE 2:**
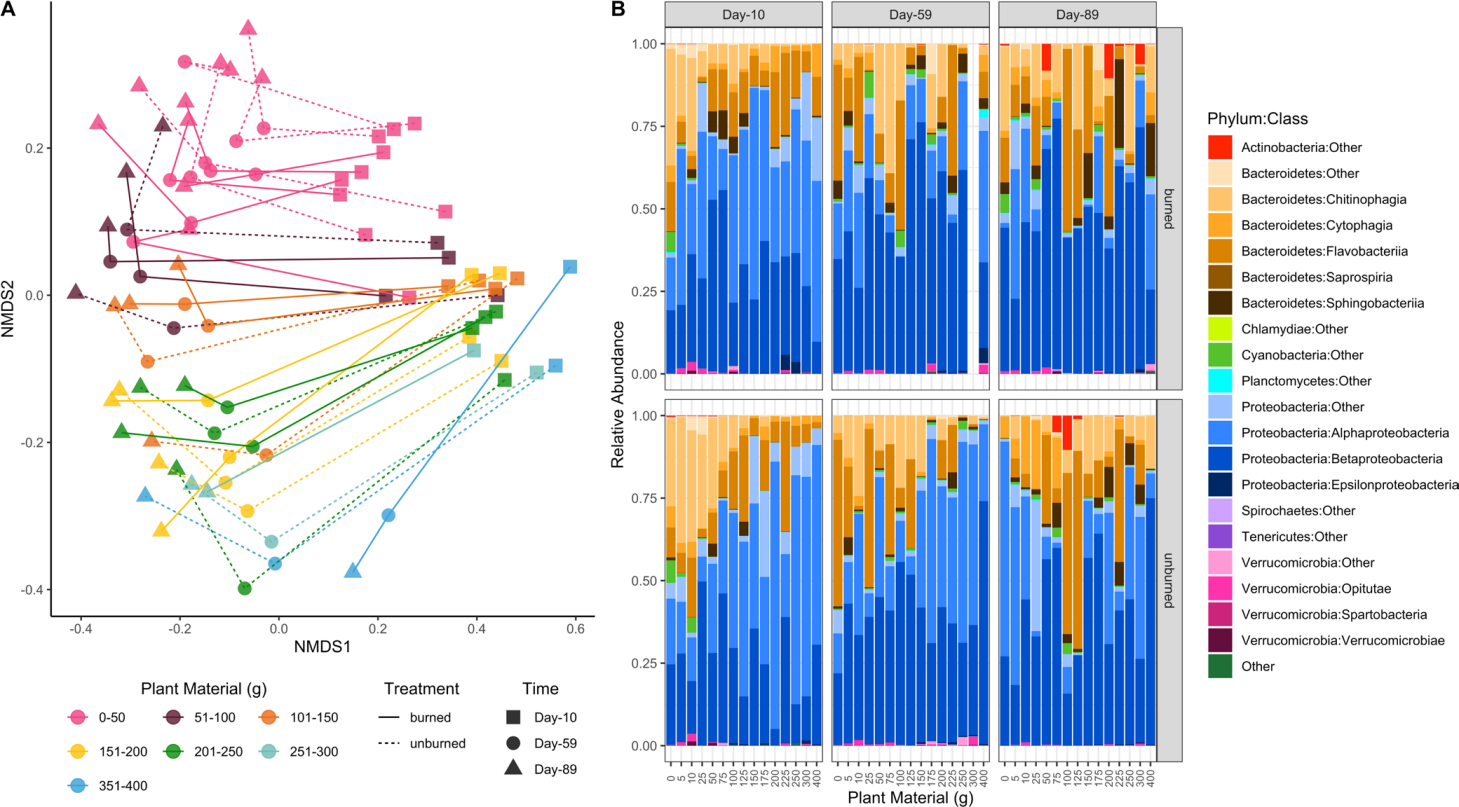
(A) Non‐metric multidimensional scaling (NMDS) plot showing variation in water microbiota over time. Each point represents a mesocosm at a single time point, and colour indicates the amount of plant mass added (in 50 g bins). Lines connect each mesocosm across time points, showing the microbiome compositional trajectory of an individual mesocosm. (B) Relative abundance of top bacterial taxa over time and treatments at the phylum and class level.

Water microbial community composition showed a consistent temporal trajectory that varied significantly with loading but not with burning treatment. Time explained 19.2%, loading explained 6.6%, and burning explained only 1.4% of changes in microbial community structure (Figure 2A; Table 1). Indeed, whether mesocosms contained ≤ 50 g of plant material explained a greater degree of microbial variation (7.1%) than accounting for the full gradient of loadings. Thresholds at 25 g and 75 g explained comparable variation (6.5% and 6.9% respectively; Table S1). Microbiomes in mesocosms containing > 100 g were largely indistinguishable from each other, and compositional dissimilarity was consistent through time, with microbial communities maintaining separation between ≤ 50 g and > 50 g mesocosms through Day‐89. Burned and unburned communities were dominated by Proteobacteria and Bacteroidetes, though the relative contributions of bacterial classes within those phyla varied over time and with loading level (Figure 2B). Alphaproteobacteria were dominant on Day‐10, while Betaproteobacteria were most abundant (particularly in burned treatments) on Days‐59 and 89. Within Bacteroidetes, the class Chitinophagia was most abundant at low loading levels on Day‐10, but their correlation with loading became more variable over time.

**TABLE 1:** PERMANOVA results for effects of experimental conditions (burning treatment, mass of plant material added, and time point) on composition of the water microbiome.

Source of variation	df	*SS*	*R* ^2^	*F*	*p*
Burning treatment	1	0.468	0.014	1.577	0.065
Plant material	1	2.239	0.066	7.553	**0.001**
Time	2	6.542	0.192	11.032	**0.001**
Residual	84	24.904	0.729		
Total	88	34.152	1.000		

*Note:* PERMANOVA table generated from 999 permutations and Bray–Curtis dissimilarity. Significant effects (*p* < 0.05) are in bold.

Abbreviations: df, degrees of freedom; *SS*, sum of squares.

**FIGURE 3:**
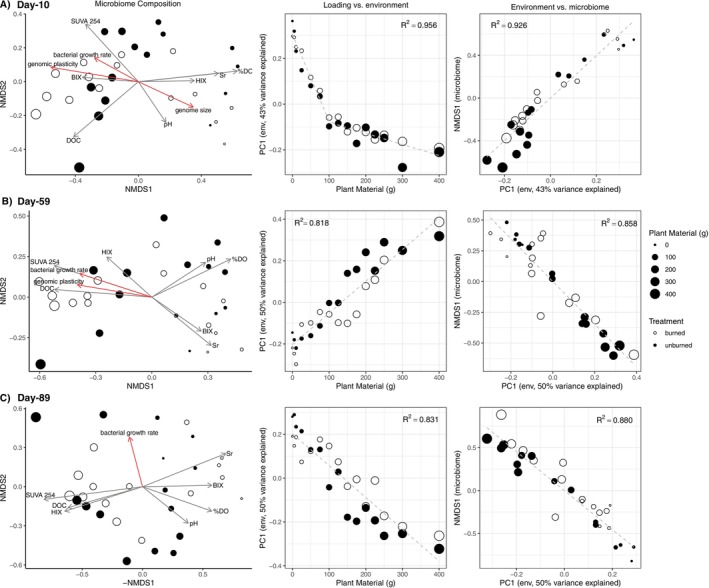
(A–C *rows*) Variation in water microbiome composition and correlations with environmental variation at Days‐10, 59, 89 respectively. Microbiome composition (*Column 1*) NMDS plots displaying variation in microbial composition, where point size indicates loading level. Vectors indicate environmental variables (*grey*) or mean bacterial genome characteristics (*red*) that correlate significantly (‘envfit’; *p* < 0.05; Table S2) with microbiome composition at each time point. X‐axis position of points and vectors in (C) are reversed (−1 × NMDS1) such that the position of high and low loading microbial communities remains consistent with (A) and (B). (*Column 2*) Loading vs. environment: correlation between plant mass and the environment using the first PCoA axis (PC1) of an environmental data ordination (see *Methods*; Figure S8). (*Column 3*) Environment vs. microbiome: correlation between environment (PC1) and water microbiome composition using the first NMDS axis (from *Column 1*). In Columns 2 and 3, dashed lines represent lines of best fit, either linear or piecewise‐linear models with one inflection point. Model selection and model fits are available in Table S3. Because correlations are between ordination axes, only model fits, not correlation slopes, are meaningful.

Specific environmental variables were correlated significantly with water microbial composition across all time points (Figure 3; Table S2). At Day‐10, HIX and dissolved oxygen were highest in low‐loading (< 100 g) mesocosms, with dissolved oxygen explaining significant compositional variation (*r* ^2^ = 0.948, *p* = 0.001). DOC, BIX, SUVA_254_, Sr., and pH were positively correlated with microbial communities in high‐loading (> 200 g) mesocosms, with DOC explaining the most microbial variation (*r* ^2^ = 0.814, *p* = 0.001). FI and temperature showed no significant correlations with microbiome composition (Table S2). At Day‐59, high HIX instead correlated with high loading levels (*r* ^2^ = 0.416, *p* = 0.001) and high BIX with low loading levels (*r* ^2^ = 0.377, *p* = 0.004), but other environmental trends (DOC, %DO, SUVA_254_, Sr., and pH) remained consistent. Day‐89 environmental trends mirrored Day‐59.

We inferred mean bacterial genome characteristics from the 16S‐ASVs and identified genomic characteristics that covaried significantly with microbial composition differences (Table S2; non‐linear relationships between loading and genome characteristics available in Figure S6). The average microbial genome size was positively correlated with loading on Day‐10 (*r* ^2^ = 0.375, *p* = 0.003). There was no significant variation in genome size at later sampling dates (Day‐59: *p* = 0.783; Day‐89: *p* = 0.783). Relative bacterial genomic plasticity was initially largest in high‐loading mesocosms and showed no significant trend on the final date (Day‐10: *r* ^2^ = 0.756, *p* = 0.001; Day‐59: *r* ^2^ = 0.462, *p* = 0.001; Day‐89: *p* = 0.098). Mean bacterial growth rate was highest in intermediate‐to‐high (200–400 g) loading mesocosms on Day‐10 (*r* ^2^ = 0.261, *p* = 0.027) and Day‐59 (*r* ^2^ = 0.498, *p* = 0.001) and lowest in intermediate‐loading mesocosms on Day‐89 (*r* ^2^ = 0.463, *p* = 0.001).

We compared loading directly to environmental variation (PC1; the first axis of an environment PCoA) and determined whether linear or piecewise‐linear models best fit the relationship (Figure 3; Table S3). Loading and environment were strongly correlated at all time points (*r* ^2^ > 0.81), and a piecewise‐linear model with a breakpoint at a plant mass of 92.19 g best fit the relationship at Day‐10 (ANOVA; F_2,26_ = 43.63, *p* < 0.001). Here, the correlation between loading level and environment also showed a threshold at approximately 100 g, with a larger degree of environmental change (steeper linear‐model slope) occurring between 0–100 g than between 125–400 g. However, piecewise‐linear models did not fit the loading‐environment relationship better than linear models without breakpoints at later time points (ANOVA; Day‐50, *p* = 0.055; Day‐89, *p* = 0.172). Environmental variation (PC1) and water‐microbiome variation (NMDS1) were also strongly correlated (all *r* ^2^ > 0.85). Linear rather than piecewise‐linear models best fit the environment‐microbiome relationship at all time points (ANOVA; Day‐10, *p* = 0.042; Day‐59, *p* = 0.156; Day‐89, *p* = 0.225).

**FIGURE 4:**
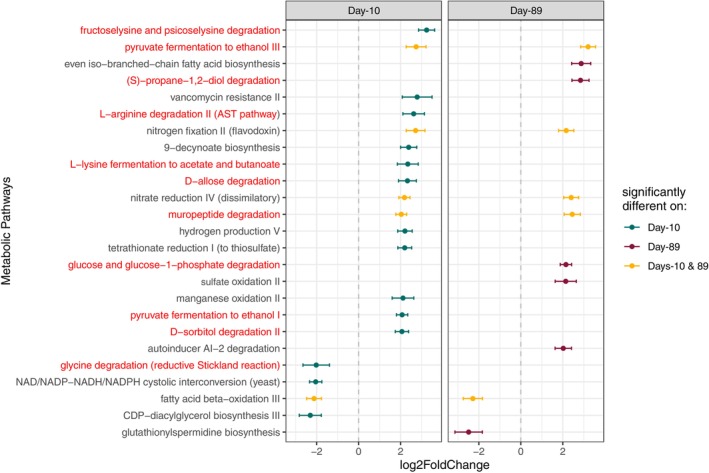
Microbial metabolic pathways (identified with paprica) in water that vary with loading level. Positive log2FoldChange indicates that a pathway increases with plant mass, and a negative log2FoldChange indicates that a pathway decreases with plant mass. Red text indicates pathways associated with carbon metabolism. Point colour indicates whether a pathway varies significantly (DESeq2; *p* < 0.01; abs(log2FoldChange) > 2) at one or both time points. Only pathways that appear in a minimum of five bacterial taxa (‘edges’, as identified by paprica) are included to limit functional changes attributable to a single bacterial taxa abundance shift. Loading level (plant mass) was centered and scaled for DESeq2 analysis (Love et al. 2014), such that a log2FoldChange of +2 indicates that the abundance of the pathway at one standard deviation above the mean loading level is 2^2^ times more abundant than that pathway at the mean loading level.

Differential abundance analysis (DESeq2) identified relative abundance shifts in water bacterial metabolic pathways across loadings, with 254 (Day‐10) and 50 (Day‐89) total pathways differing (Wald test; *p* < 0.01) across loadings. When only those pathways associated with a minimum of five bacterial taxa and for which abs(log2FoldChange) > 2 were included, we found 25 total pathways varied significantly, with 14 varying only on Day‐10, 6 varying only on Day‐89, and 5 varying significantly at both times (Figure 4). Day‐10 pathways primarily included carbon‐substrate degradation pathways that increased with loading level. Despite the increase in aromaticity with loading, there was no statistically significant selection for pathways associated with the breakdown of aromatic compounds (as determined by MetaCyc) at high loadings. Overall, burning‐treatment explained no significant variation in water bacterial metabolic pathway abundance, while loading explained 2.9% and time explained 6.9% of pathway variation (Table S16).

**FIGURE 5:**
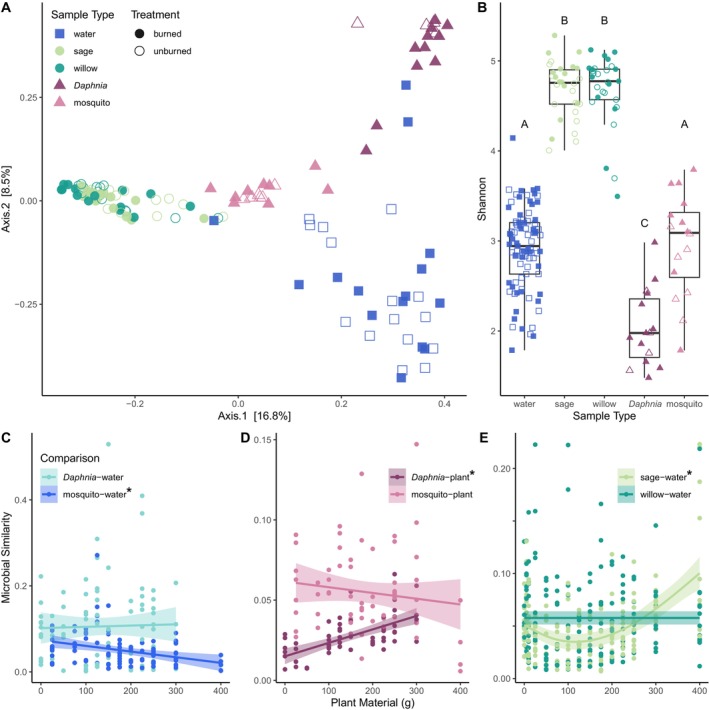
(A) Principle coordinate analysis (PCoA) of variation in water, plant (sage and willow), and zooplankton (*Daphnia* and mosquito larvae) associated microbiomes at the final sampling point for each. (B) Microbial alpha diversity (Shannon) of each sample type. Letters indicate significantly different groups (multiple pairwise Wilcoxon‐rank sum tests; *p* < 0.01). Colour represents sample type, and treatment is indicated by closed versus open shapes. (C) Plankton (*Daphnia* and mosquito) microbiomes compared to water microbiomes; (D) zooplankton microbiomes compared to plant microbiomes; and (E) plant microbiomes compared to water microbiomes across plant loading levels at the final time point. Microbial similarity was quantified as 1—Bray–Curtis dissimilarity between paired individual samples at the same loading level. Linear regression lines were fit in (A) and (B), and a generalised additive model was fit in (C) due to the non‐monotonic trend (Table S4). Significant relationships are denoted with asterisks. Models were not separated by plant species in (D) because there was no significant difference between willow and sage comparisons to each zooplankton group. Comparisons between and within treatments (burned vs. burned, burned vs. unburned, etc.) were modelled collectively, as effects of burning were minor (Figure S1; Tables S5, S6).

To address variability in long‐term response of different microbial community types to disturbance, we compared microbial communities across water samples, plant detritus, and zooplankton hosts three months post‐disturbance. Samples showed strong differentiation between water‐, zooplankton‐, and plant‐associated microbiomes (Figure 5A). Indeed, sample source explained the most microbial variation (20.8%), with burning treatment (0.9%) and loading (3.8%) contributing minimally (Table 2). While *Daphnia* and mosquito microbiomes showed clear differentiation, plant‐associated microbiomes did not differ between sage and willow detritus. Sample types also showed distinct alpha diversity trends; plant‐associated microbiomes were more diverse than other microbiomes, and mosquito microbiomes were more diverse than *Daphnia* microbiomes (Figure 5B). Across loading levels, *Daphnia* microbiomes were consistently more similar to water microbial communities, while mosquito microbiomes were more similar to plant‐associated microbiomes (Figure 5C,D; Table S4). As loading increased, mosquito microbiomes showed decreased similarity to water, and *Daphnia* microbiomes increased in similarity to plant microbiomes. Similarity between plant and water microbiomes showed no consistent trend with loading (Figure 5E).

**TABLE 2:** PERMANOVA results for effects of sample source (water, plant detritus, zooplankton), within‐source sample type (plant species, zooplankton species), and experimental conditions (burning treatment, mass of plant material added) on the composition of the microbiome at the final sampling time point.

Source of variation	df	*SS*	*R* ^2^	*F*	*p*
Sample source	2	10.261	0.208	17.797	**0.001**
Sample type	2	4.092	0.083	7.098	**0.001**
Burning treatment	1	0.466	0.009	1.615	**0.040**
Plant material	1	1.863	0.038	6.462	**0.001**
Residual	113	32.576	0.661		
Total	119	49.259	1.000		

*Note:* PERMANOVA table generated from 999 permutations and Bray–Curtis dissimilarity. Significant effects (*p* < 0.05) are in bold.

Abbreviations: df, degrees of freedom; *SS*, sum of squares.

### 3.2 Zooplankton Community Composition and Densities

**FIGURE 6:**
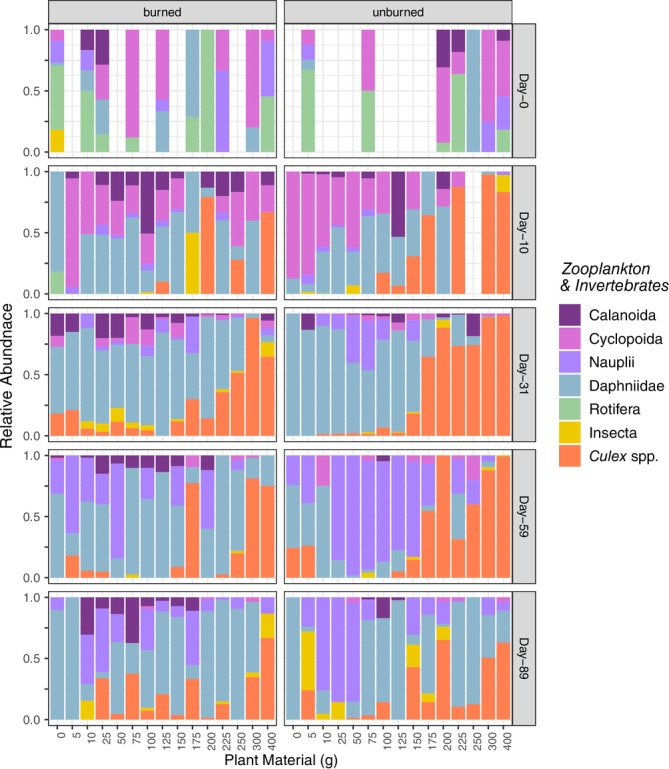
Relative abundance of zooplankton community members (planktonic crustaceans and immature macroinvertebrates) in burned and unburned experimental mesocosms on Days 0, 10, 31, 59, and 89.

Zooplankton communities showed distinct trends associated with both loading and burning treatment through time (Figure 6). Burning treatment explained 2.3%, loading explained 10.3%, and time explained 24.9% of zooplankton community variation (Table S15). Generally, Daphniidae (followed by Cyclopoida and Copepod nauplii) were the most abundant members of the zooplankton community at all time points. However, Daphniidae declined and mosquitoes (*Culex* sp.) increased with greater loading in the burned and unburned treatments, and this effect was most pronounced in unburned mesocosms. Daphniidae densities showed negative (Day‐10) or unimodal relationships (Days‐31 and 89) with loading, generally increasing with plant biomass until intermediate‐loading (100–200 g) and declining thereafter (Figure S9; Tables S7 and S8). Daphniidae densities were lowest at Day‐59 and were significantly greater in burned relative to unburned mesocosms at intermediate‐loading (100–250 g). Conversely, Copepoda zooplankton showed negative linear relationships with loading and were not affected by burning, except at Day‐31 where a unimodal relationship and two‐fold greater abundance in low‐loading (50–125 g) relative to burned mesocosms was observed. In contrast, mosquito larvae densities increased with loading in intermediate‐to‐high mesocosms (> 200 g) and were significantly higher in high loading unburned treatments. This resulted in a 10‐fold increase in mosquito larvae in unburned mesocosms (400 g, Day‐31). By Day‐89, mosquito larvae densities had declined, but remained higher in unburned mesocosms receiving > 250 g of plant detritus.

### 3.3 Flow Cytometry and Greenhouse Gas δ^13^C

Cell densities were primarily influenced by time (peaking at Day‐10) and showed inconsistent relationships with burning and loading at each time point. Autofluorescent cells (photosynthetic autotrophs) were highly variable at Day‐10 (0.2–4.0 × 10^6^ cells) and did not differ by loading or burning treatment through Day‐59 (Figure S2; Tables S9 and S10). At Day‐89, significant loading and burning effects were observed, with greater autotrophic cell abundance in unburned, intermediate‐loading mesocosms (125–225 g). Total cell abundance (i.e., Syber Green) was affected by loading, being higher at 250–350 g mesocosms early on (Day‐10). By Day‐89, a positive linear relationship between loading and cell abundance was observed in unburned mesocosms, with no relationship in burned mesocosms (Figure S2).

δ^13^CO_2_ values showed an exponential decline with loading (Days‐10 and 31) through Day‐59, with δ^13^CO_2_ values then being significantly higher in intermediate‐loading burned treatments (60‐175 g). Similarly, on Day‐59, significant effects of loading and burning were observed, with burned mesocosms showing greater δ^13^CH_4_ variability and lower δ^13^CH_4_ values at low loadings (< 25 g) (Figure S4; Tables S11 and S12).

## 4 Discussion

We tested the effects of post‐fire terrestrial deposition in aquatic ecosystems, predicting that manipulating the quality (burning) and quantity (loading) of terrestrial material would yield initially distinct ecosystem states that maintained differences over time. We found water microbial community differences were primarily driven by loading with little burning‐treatment effects. Loading effects on water microbial communities revealed a threshold between 25 g and 100 g of plant material where water communities transitioned to new compositional states. The rate of environmental change across loading levels also highlighted a threshold at ~100 g on Day‐10, where increasing the amount of plant material within the 0–100 g loading range resulted in far more environmental turnover than adding the same plant mass within the 125–400 g range. We hypothesise that this threshold represents a division between nutrient regimes characterised by changes in DOM composition and bacterioplankton communities' response to changing water chemistry and productivity. For instance, changing DOM composition can shift lake bacterial community structure and production (Judd et al. 2006), and low oxygen decreased bacterial richness and altered community composition in a seasonally hypoxic estuary (Spietz et al. 2015). However, initial non‐linear effects of loading quantity on mesocosm environmental conditions transitioned to linear effects over time, suggesting that microbial processes eventually metabolised organic material in high loading treatments, decreasing threshold responses. Given that microbial communities and mesocosm environmental conditions demonstrated a similar threshold response to loading respectively, it follows that the relationship between microbiome composition and environmental conditions was, in turn, linear. This indicates that loading‐dependent environmental shifts had regular, stepwise effects on microbial community shifts. High rates of functional redundancy and plasticity among microbes (Comte et al. 2013) may have contributed to this linear response of microbes to overall environmental shifts, such that microbes respond to small shifts in environmental conditions with a combination of species replacement and plasticity that moderated total community turnover.

Plant loading had profound impacts on tank environments by increasing dissolved organic carbon [DOC] and inorganic nutrients (notably total phosphorus (Wall et al. 2024)), leading to declines in dissolved oxygen (50% decrease in DO between 100 g and 0 g, Day‐10) and attenuated microbial decomposition of DOC as loading increased (Spiegel et al. 2024). We found water microbial community composition varied along expected [DOC] and %DO gradients, as well as with aromaticity (SUVA_254_) and organic matter type (BIX and HIX). Some bacterial taxa trends reflected this environmental gradient; bacteria within the Chitinophagia class, for example, are typically aerobic (Kirchman 2002) and were most abundant at low‐loading, early time points, when DO showed a consistent decreasing trend with loading level. However, peaks in BIX and bacterial cell densities between 200–300 g loadings on Day‐10 also indicate that loading may have stimulated heterotrophic bacterial production and carbon mineralisation (Vidal et al. 2011; Berggren et al. 2023) through respiration and transformation of aromatic compounds to new labile compounds, despite low carbon use efficiency (Fasching et al. 2014). Thus, loading effects on microbial community structure may be contingent on the interaction between environmental thresholds (such as %DO) and DOM composition. Future examination of specific microbial taxa under these varying %DO and DOM conditions could elucidate whether the success of particular groups is due to the ability to tolerate hypoxic conditions and/or to assimilate compounds of terrestrial origin in support of production.

As microbial community composition shifted across the loading gradient on Day‐10, so did characteristics of the average community‐level predicted bacterial genomes. Increased mean bacterial growth rates and decreased genome size were associated with high‐loading mesocosms. Hypoxic conditions under high‐loading may promote the growth of microbes with streamlined genomes that reduce their metabolic costs (Giovannoni et al. 2014; Simonsen 2022), while increased [DOC] supports increased growth rates. Relative genomic plasticity, which represents increasing divergence of microbial genomes from the marker gene phylogeny, also increased with loading. Together these trends suggest a potential selection for streamlined genomes and functional specialisation occurring in stressful high‐loading environments, consistent with genomic trends observed in stressed soil (Simonsen 2022) and marine (García‐Fernández et al. 2004) systems. The correlation between mean bacterial genome traits and microbial community variation across loading levels further indicates that loading drives compositional shifts favouring net growth of bacteria with specific genome types best adapted to the unique environmental conditions associated with resource quality and quantity.

Predicted bacterial metabolic pathway enrichment also showed consistent changes in relation to the loading treatments at the initial and final time points. On Day‐10, several enzymatic degradation pathways, primarily associated with the breakdown of carbon sources (e.g., fructolysine and psicoselysine, D‐allose, D‐sorbitol) increased significantly with loading, while only four enzymatic pathways (primarily involving general cell functions such as electron transfer) were enriched at low‐loadings. Labile compound availability can determine the mineralization of recalcitrant compounds (‘priming effect’) (Guenet et al. 2010); thus, despite an increase in recalcitrant carbon at high‐loading levels, the simultaneous increase in labile carbon may have promoted microbial degradation activity. More carbon resources (both labile and recalcitrant) initially available for microbes to degrade in high‐loading mesocosms may have broadened the range of enzymatic degradation pathways that microbes can use.

Isotope analysis of greenhouse gasses provided further evidence of loading effects on organic compound degradation. We observed higher δ^13^CO_2_ values in low‐loading treatments, indicative of greater photosynthesis rates (Farquhar et al. 1989; Metya et al. 2021). In addition, the relatively high δ^13^CH_4_ values (~ −48‰ to −45‰) indicate that high [CH_4_] (9‐times above ambient air) reported in a companion study (Wall et al. 2024) was likely not driven by anaerobic methanogenesis (δ^13^CH_4_ of −110‰ to −50‰ [Schaefer et al. 2016; Perez‐Coronel and Beman 2022]), but rather oxic methane production by phototrophic bacteria or cyanobacteria (Perez‐Coronel and Beman 2022). Together, these findings suggest that increased plant deposition may decrease photosynthesis, while also highlighting the potential contribution of oxic methane production in freshwater systems receiving terrestrial material (Bižić et al. 2020; Grossart et al. 2011). Considering both δ^13^CO_2_ and δ^13^CH_4_ demonstrated no differences between burned and unburned treatments across loading levels until Day‐89, accumulated effects of burning on bacterial metabolism over time appear minor.

Overall, microbial community composition remained distinct across loadings over time, while environmental conditions and inferred bacterial genome characteristics became more similar. Total bacterial biomass normalised across loading levels (though showed minor treatment effects by the final time point). These results suggest that as the environment ([DOC], %DO) stabilises, environmental selection of microbes with specific genome characteristics may decrease, resulting in bacteria with comparable ecological strategies (Bell and Bell 2020). The total number of metabolic pathways that varied significantly with loading level also decreased between the initial and final sampling points, potentially indicating decreasing functional diversity over time. Despite the extreme response of environmental conditions to loading > 100 g—notably an exponential increase in [DOC] and concomitant hypoxia—time decreased the non‐linear loading‐by‐environment relationship, reducing initial threshold shifts in microbial communities post‐deposition. These patterns indicate that while initial disturbance has long‐term effects on community composition, functional differences and community‐level contributions to ecosystem function slowly converge across mesocosms as microbial respiration reduces [DOC].

Like microbial communities, zooplankton abundances and community composition were strongly influenced by loading levels, with community composition shifting at ~100–150 g. Absolute abundance of the crustaceans Daphniidae and copepods declined at higher loading levels at the first time point, and mosquito larvae dominated in relative abundance, a trend further amplified in burned treatments over time. Both *Daphnia* and mosquito larvae are filter feeders typically consuming phytoplankton, detritus, and bacterioplankton (Akbar et al. 2022; Strand 2018), and therefore will both be affected by food quality and quantity. However, browning reduced *Daphnia* reproduction due to lack of nutritious algal resources (Taipale et al. 2019), and *Daphnia* are more sensitive to low %DO compared to mosquito larvae (Nebeker et al. 1992; Silberbush et al. 2015), which primarily breathe atmospheric air at the water surface and use DO facultatively (Clements 1992). Differences in tolerance of environmental conditions (primarily oxygen levels) likely supported mosquito dominance over *Daphnia* and copepods in high loading tanks. The altered chemical composition of burned relative to unburned materials and decreasing food quality exacerbated this effect. Resource competition between *Daphnia* and mosquitoes has been shown to decrease mosquito survival (Thakur and Kocher 2022). Thus, an environment‐driven decrease in *Daphnia* populations may reduce direct competition and support mosquito dominance.

As filter feeders, both zooplankton groups have internal microbiomes strongly influenced by the environment (Akbar et al. 2022; Strand 2018; Duguma et al. 2013). However, *Daphnia* and mosquito larvae microbiomes were clearly distinct both compositionally and in terms of alpha diversity, suggesting that the intake of environmental microbes and/or internal host conditions differently shaped their microbiomes in a manner that may have contributed to fitness and observed population abundances. Notably, *Daphnia* microbiomes consistently exhibited greater similarity than mosquito microbiomes to the water microbiome at the final time point, suggesting that *Daphnia* may be less capable of internal microbial selection or filtering than mosquito larvae. If harmful or insufficient beneficial microbes exist in a disturbed water microbiome from which *Daphnia* establish their internal microbiome, *Daphnia* survival may decrease (Hegg et al. 2021; Akbar et al. 2021). While both *Daphnia* and mosquito larvae acquire their microbiomes from multiple sources (i.e., vertical (germline) and horizontal (environment) transmission) (Akbar et al. 2022; Schrieke et al. 2022), we found mosquito larvae microbiomes were more distinct from the environmental community (Strand 2018) than crustacean zooplankton microbiomes, reducing the potential impact of a disturbed water microbiome on mosquito host‐associated microorganisms.

Our results indicate that wildfire‐driven terrestrial loading has significant effects on biological communities across multiple trophic levels within aquatic ecosystems. Loading‐driven effects on environmental conditions, chemistry (%DO, DOM), and microbial activity (Zheng et al. 2022) were more significant in shaping microbial community composition and function than chemical differences between burning treatments (Thuile Bistarelli et al. 2021). Loadings produced initial threshold responses of environmental conditions and microbiome composition, but these differences diminished over time. Zooplankton communities were shaped by fire and loading effects, with high‐loading and unburned treatments being dominated by insects (mosquitos) over zooplankton crustaceans. Overall, we found that microbial and zooplankton community composition retained loading‐disturbance effects, but ecosystems maintained only minor environmental and predicted functional differences, though some variation in microbial community‐level function was persistent across the most divergent disturbances (i.e., comparing highest and lowest loading levels).

While we found that aquatic communities are capable of recovering some environmental conditions and ecosystem function following small‐scale, experimental wildfire disturbances, the ecological impacts of wildfires on terrestrial and aquatic habitats can extend for years (Bixby et al. 2015; McCullough et al. 2019). As global temperatures rise and the frequency and severity of wildfires increase (Jones et al. 2022; Liu et al. 2010), aquatic ecosystems may experience greater inputs of pyrogenic materials (e.g., ash, aerosols, debris run‐off) in the coming decades. These disturbances are increasingly likely to exceed the loading thresholds that we identified, leading to shifts in the community composition and function of microorganisms that may persist and accumulate over time, manifesting in long‐term consequences for aquatic ecosystem function.

Although our experimental approach offers a controlled setting to observe microbial responses, it cannot fully capture the complexity and heterogeneity of natural watersheds affected by wildfire. Burn extent and fire severity, as well as watershed‐specific factors such as slope and vegetation types and rural versus urban pyrogenic material, may influence microbial community shifts in ways that are difficult to replicate in mesocosms, and fire‐driven changes are likely to extend beyond 90 days (Bixby et al. 2015). Our findings on microbial genome characteristics and metabolic pathways are also subject to the limitations of functional genome databases for 16S‐sequencing data. Nevertheless, we identify functionally relevant community‐level genome shifts that, together with our microbial community time series analyses, can serve to motivate further hypothesis testing of wildfire effects on aquatic ecosystems and microorganisms. Future metagenomics sequencing and microbial culturing work that aims to identify genomic changes in specific aquatic taxa and link them to functional shifts could improve our understanding and prediction of aquatic ecosystem responses to wildfire disturbance.

## Author Contributions

C.B.W., C.J.S., and J.S.B. conceived of and designed the project. C.B.W., C.J.S., E.M.D., and M.G.P. set up the experiment and conducted field sampling. C.B.W., C.J.S., E.M.D., and M.G.P. conducted lab work in J.B.S.'s laboratory at UC San Diego, with methodological input from E.P.‐C. and S.L.J. M.Y.D., C.B.W., N.E., and J.S.B. conducted bioinformatics and data analyses. M.Y.D. and C.B.W. designed figures and drafted the manuscript, with input from S.L.J., J.S.B., J.B.S., and other co‐authors. All co‐authors read and approved the final manuscript.

## Conflicts of Interest

The authors declare no conflicts of interest.

## Supporting information


**Data S1.** Supporting Information.

## Data Availability

All data and scripts for the paprica pipeline and downstream analyses are available at Github (https://github.com/bowmanjeffs/paprica; http://www.github.com/mdemmel/Pyro‐microbes) and are archived at Zenodo (https://doi.org/10.5281/zenodo.15399887). Sequence data were submitted to the NCBI SRA at BioProject PRJNA1092644.
