# Supplementary material for: Wildfire‐Driven Changes in Terrestrial Subsidies Shift Freshwater Microbial and Zooplankton Communities to New Compositional States
Source: Mol Ecol. 2025 May 20;34(20):e17794. doi: 10.1111/mec.17794 (PMC12530278; doi:10.1111/mec.17794)
Supplement: Supplementary file 1 — Data S1. Supporting Information. [file MEC-34-e17794-s001.pdf]

Supplemental Materials

Wildfire-driven changes in terrestrial subsidies drive freshwater microbial and zooplankton communities to new compositional states

Margaret Y. Demmel‡, Christopher B. Wall‡, Cody J. Spiegel, Natalia Erazo, Evelyn M. Diaz, Madeline G. Perreault, Elisabet Perez-Coronel, Sara L. Jackrel, Jeff S. Bowman, Jonathan B. Shurin

‡ = both authors contributed equally and share first authorship

Corresponding author: Margaret Y. Demmel

Email: mdemmel@ucsd.edu

This PDF file includes:

Supplemental Materials and Methods

Supplemental Table S1 to S16

Supplemental Figures S1 to S10

Supplemental References

Supplemental Materials and Methods

Sampling design and response metrics

Twenty-three sage plants were purchased (9 June 2021) and grown in a 1:1 soil:vermiculite mixture in pots for 60 days at the University of California San Diego Biology Field Station (La Jolla, CA) until harvest on 9 September 2021. Willow plant material was collected from the University of California Dawson Los Monos Reserve in Buena Vista, CA, on 6 October 2021. Harvested sage and willow leaves and stems (< 2 cm diameter) were kept separate, air dried in a greenhouse (24 h) and a drying oven (24 h at 45°C). Dried plant material was cut (<10 cm) and either remained unburned or were exposed to varying degrees of burning at low and high burn severity to mimic non-uniform effects of fire on plant material in nature. Plant material (leaves and stems) were flamed briefly with a handheld butane torch in 75 L aluminum containers. Burned plant materials were pooled according to plant species with a cumulative biomass of ~2 kg of burned and unburned material for both sage and willow.

We measured dissolved oxygen (%DO) at dawn on each sampling day using a YSI Pro-Plus water sensor (YSI Inc., Yellow Spring, OH) calibrated against certified standards. Water samples were collected, and filtered (0.7 µm GF/F, pre-combusted 550˚C, 2h) into pre-combusted borosilicate amber vials, with unacidified samples used for excitation-emission matrix spectral analysis and acidified samples (37% HCl, pH 3) to quantify DOC.

16S rRNA gene sequencing

The V4 region of the 16S rRNA gene was amplified using modified primers 515F (5'-GTGYCAGCMGCCGCGGTAA-3') and 806R (5′-GGACTACNVGGGTWTCTAAT-3′), which increase aquatic microbial taxa detection [1]. Primers included Illumina flowcell adaptor sequences [2] and a 12bp barcode sequence on the forward primer to allow sample pooling. 25 µL PCR reactions contained 9.5 µL of MO BIO PCR Water (Certified DNA-Free), 12.5 µL of QuantaBio's AccuStart II PCR ToughMix (2x concentration), 1 µL Golay barcode tagged Forward Primer (5 µM concentration), 1 µL Reverse Primer (5 µM concentration), and 1 µL of template DNA. DNA was amplified with the following thermocycling conditions: 94˚C for 3 minutes; 35 cycles of 94˚C for 45 s, 50˚C for 60 s, and 72˚C for 90 s; and 72˚C for 10 minutes. Amplified samples were quantified using PicoGreen (Invitrogen), and samples were then pooled in equimolar volumes. Finally, the pooled amplicons were cleaned (AMPure XP Beads; Beckman Coulter), quantified (fluorometer; Qubit, Invitrogen), and diluted before being sequenced on a 151x151 bp paired-end Illumina MiSeq run at Argonne National Laboratory.

Carbon dioxide and methane stable isotope carbon analysis

$\delta^{13}CO_2$ was analyzed on a Thermo Scientific GasBench II coupled to a Thermo Finnigan Delta Plus XL isotope-ratio mass spectrometer. $\delta^{13}CH_4$ was analyzed on a Thermo Scientific GasBench II + PreCon trace gas concentration system coupled to a Thermo Scientific Delta V Plus isotope-ratio mass spectrometer at the University of California Davis. A pure CO_2 reference gas was used to calibrate provisional $\delta^{13}C$ values of the sample peaks. The isotopic values were then adjusted for changes in linearity and instrumental drift using secondary reference materials before the measurements were scale-normalized to the primary reference material using three pure CO_2

secondary reference materials, calibrated against certified standard reference materials available from NIST and IAEA, with $\delta^{13}C$ expected measurement error ± 0.20 ‰.

Flow Cytometry

We ran samples on a Guava easyCyte 11HT (Luminex, Austin, TX, USA) using both a blue (488 nm) and violet (405 nm) laser and evaluated the fluorescence signals of unstained chlorophyll-containing samples using autofluorescence, measuring forward scatter, side scatter, red emission (40 5nm excitation/695 nm emission), and yellow emission (488 nm excitation/583 nm emission). We then stained samples with SYBR Green I (SG-stained; ThermoFisher Scientific, Waltham, MA, USA) at the manufacturers recommended working concentration to visualize all cells with double-stranded DNA, measuring forward scatter, side scatter, and green emission (488 nm excitation/525 nm emission) [3]. To determine absolute cell counts, we spiked a standard volume of 1:10 diluted 123-count beads (ThermoFisher Scientific) to each sample and blank.

GAM model selection

Within each time point, we applied a model selection framework that compared three GAMs: a model consisting of a single global smoother fit to all data; a model with a global smoother and a parametric 'Treatment' term, allowing different intercepts for fire treatments (burned vs. unburned); and a model with a factor-smooth term that fit different smoothers for each fire treatment along with a parametric term for treatment-specific intercepts. We compared candidate models using Akaike Information Criterion (AIC), selecting models with the lowest AIC. GAMs were inspected for model concurvity using the 'concrvity' function in gratia [4]. 'Gam.check' was used to assess model fits, and 'anova.gam' generated ANOVA tables in the mgcv package.

Differences between burned and unburned treatments were assessed by calculating the "differences smooth" of two conditions (i.e., burned and unburned smooths) in tidymv [5] and determining regions of significance where s(treatment 1) - s(treatment 2) is greater than zero and does not include treatment-smooth confidence intervals.

Supplemental Tables

Table S1. Comparison of PERMANOVA results for effects of plant mass added at different threshold cutoffs between 5g and 300 g on microbiome composition across all time points and treatments.

Cutoff value (plant mass ≤)	*SS*	R^2	*F*	*p-value*
0g	0.692	0.020	1.800	0.021
5g	1.278	0.037	3.381	0.001
10g	1.749	0.051	4.696	0.001
25g	2.217	0.065	6.038	0.001
**50g**	**2.417**	**0.071**	**6.627**	**0.001**
75g	2.340	0.069	6.400	0.001
100g	2.074	0.061	5.626	0.001
125g	2.007	0.059	5.431	0.001
150g	1.751	0.051	4.701	0.001
175g	1.539	0.045	4.104	0.001
200g	1.292	0.038	3.421	0.001
225g	1.161	0.034	3.062	0.002
250g	0.972	0.028	2.549	0.003
300g	0.658	0.019	1.709	0.037

PERMANOVA results generated from 999 permutations and Bray-Curtis dissimilarity, where the source of variation was a binary condition based on the plant mass cutoff value. For example, for the cutoff value of 50g, the explanatory variable was coded as 0 for plant mass amounts ≤50g and 1 for plant mass amounts >50g. *SS* = sum of squares. Row with the maximum R^2 value is in bold.

Table S2. Results of fitting nine environmental and three inferred bacterial genome characteristic vectors to microbial composition NMDS ordinations at each time point.

Time	*Variable Type*	*Variable*	R^2	*p-value*
Day-10	Environmental	%DO	0.948	**0.001**
		DOC	0.814	**0.001**
		FI	0.131	0.161
		BIX	0.306	**0.006**
		HIX	0.293	**0.008**
		$SUVA_{254}$	0.707	**0.001**
		Sr	0.609	**0.001**
		Temp	0.047	0.495
		pH	0.292	**0.015**
	Bacterial Genome	mean genome size	0.375	**0.003**
		mean genomic plasticity	0.756	**0.001**
		mean growth rate	0.261	**0.027**
Day-59	Environmental	%DO	0.723	**0.001**
		DOC	0.757	**0.001**
		FI	0.071	0.390
		BIX	0.377	**0.004**
		HIX	0.416	**0.001**
		$SUVA_{254}$	0.911	**0.001**
		Sr	0.650	**0.001**
		Temp	0.061	0.471
		pH	0.421	**0.001**
	Bacterial Genome	mean genome size	0.019	0.783
		mean genomic plasticity	0.462	**0.001**
		mean growth rate	0.498	**0.001**
Day-89	Environmental	%DO	0.530	**0.001**
		DOC	0.639	**0.001**
		FI	0.160	0.105
		BIX	0.424	**0.001**
		HIX	0.567	**0.001**
		$SUVA_{254}$	0.883	**0.001**
		Sr	0.810	**0.001**
		Temp	0.023	0.720
		pH	0.425	**0.001**
	Bacterial Genome	mean genome size	0.018	0.783
		mean genomic plasticity	0.160	0.098
		mean growth rate	0.463	**0.001**

Vectors were fit using the 'envfit' function in vegan. Variables with p-values < 0.05 (bolded) were considered significant covariation and included in NMDS plots (**Fig. 3; column 1**). Variables are defined in *Methods*.

Table S3. Results of model fits and comparisons of linear and piecewise-linear models for the effect of plant mass on environment (quantified as principal component 1 (PC1); the first axis of an environmental ordination (env)), and the effect of environment (PC1) on the water-associated microbiome (quantified as non-metric multidimensional scaling axis 1 (NMDS1); the first axis of a microbiome ordination (mb)).

Time	*Comparison*	*Model Type*	*df*	*F*	R^2	*p-value*
Day-10	PC1 (env) ~ plant mass	linear	1, 28	133.22	0.820	< 0.001
		segmented	3, 26	208.71	0.956	< 0.001
	anova (linear, segmented)		2, 26	43.63	*NA*	**< 0.001**
	NMDS1 (mb) ~ PC1 (env)	linear	1, 28	365.70	0.926	< 0.001
		segmented	3, 26	146.79	0.938	<0.001
	anova (linear, segmented)		2, 26	3.58	*NA*	0.0422
Day-59	PC1 (env) ~ plant mass	linear	1, 27	127.57	0.818	< 0.001
		segmented	3, 25	51.60	0.844	< 0.001
	anova (linear, segmented)		2, 25	3.26	*NA*	0.055
	NMDS1 (mb) ~ PC1 (env)	linear	1, 27	170.57	0.858	< 0.001
		segmented	3, 25	62.43	0.868	0.025
	anova (linear, segmented)		2, 25	2.01	*NA*	0.156
Day-89	PC1 (env) ~ plant mass	linear	1, 28	143.81	0.831	< 0.001
		segmented	3, 26	52.23	0.841	< 0.001
	anova (linear, segmented)		2, 26	1.89	*NA*	0.172
	NMDS1 (mb) ~ PC1 (env)	linear	1, 28	213.23	0.880	< 0.001
		segmented	3, 26	75.08	0.885	< 0.001
	anova (linear, segmented)		2, 26	1.58	*NA*	0.225

NMDS1 (mb) values were extracted from **Fig 3**. PC1 (env) values were extracted from **Fig S8**; environment principal coordinate analyses (PCoAs) were run using nine environmental variables (described in *Methods*; **Table S2**). Separate ordinations were run, and axes extracted at each time point. Basic linear models (denoted as linear in *Model Type*) and piecewise linear models with one unknown inflection point (denoted as segmented in *Model Type*) were run for each comparison. The two models were then compared using an ANOVA test, where a p-value < 0.01 indicated that the segmented model was a better fit (bolded).

Table S4. Results of model fits for effect of plant mass on pairwise microbial similarities (1-Bray-Curtis) by sample type comparisons.

Model Call	*Comparison*	*df*	*F*	*Adj r-squared*	*p-value*
lm	*Daphnia*-water	87	0.072	-0.011	0.789
	mosquito-water	92	10.83	0.096	**0.001**
	Daphnia-plant	56	35.75	0.379	**<0.001**
	mosquito-plant	62	1.178	0.003	0.282
gam	willow-water	170	*NA*	-0.006	0.900
	sage-water	176	*NA*	0.088	**<0.001**

lm refers to linear model fits, *gam* refers to non-linear generalized additive model fits. All models are testing effects of plant mass on water-zooplankton, zooplankton-plant, or plant-water microbial similarity, thus only comparisons within mesocosms (where the same plant mass was added) are included. Model call determination was based on visual assessment of points and plotted in **Fig 5**. Significant effects ($p<0.05$) are in bold.

Table S5. PERMANOVA results for effects of experimental conditions (burning treatment, mass of plant material added) and plant species (sage or willow) on composition of the plant-associated microbiome at the final sampling time.

Source of Variation	*df*	*SS*	R^2	*F*	*p-value*
Burning Treatment	1	0.474	0.025	1.711	**0.025**
Plant Material	1	2.189	0.116	7.889	**0.001**
Plant Species	1	0.928	0.049	3.346	**0.001**
Residual	55	15.246	0.809		
Total	58	18.835	1.000		

PERMANOVA table generated from 999 permutations and Bray-Curtis dissimilarity. *df*=degrees of freedom; *SS*=sum of squares. Significant effects (p<0.05) are in bold.

Table S6. PERMANOVA results for effects of experimental conditions (burning treatment, mass of plant material added) and zooplankton taxa (*Daphnia* or mosquito larvae) on composition of the zooplankton-associated microbiome at the final sampling time.

Source of Variation	*df*	*SS*	R^2	*F*	*p-value*
Burning Treatment	1	0.624	0.058	2.527	**0.019**
Plant Material	1	0.576	0.054	2.332	**0.028**
Zooplankton Taxa	1	2.889	0.269	11.796	**0.001**
Residual	27	6.663	0.620		
Total	30	10.751	1.000		

PERMANOVA table generated from 999 permutations and Bray-Curtis dissimilarity. *df*=degrees of freedom; *SS*=sum of squares. Significant effects (p<0.05) are in bold.

Table S7. Model selection for zooplankton (Daphniidae, Copepoda) and mosquito (*Culex* spp.) densities with candidate GAM models* assessed at four time points post addition of plant material to experimental mesocosms.

Taxa	*Time*	*Model*	*df*	*AIC*	*ΔAIC*
Daphniidae	Day-10	~Treatment + s(plant material, by=Treatment)	5.0	195.4	
		~Treatment + s(plant material)	4.0	193.8	
		~s(plant material)	3.0	**191.8**	0.0
	Day-31	~Treatment + s(plant material, by= Treatment)	9.6	256.6	
		~Treatment + s(plant material)	6.4	257.5	
		~s(plant material)	5.5	**255.6**	0.0
	Day-59	~Treatment + s(plant material, by= Treatment)	6.8	**162.5**	-9.3
		~Treatment + s(plant material)	5.2	163.9	
		~s(plant material)	3.8	171.7	
	Day-89	~Treatment + s(plant material, by= Treatment)	7.1	272.7	
		~Treatment + s(plant material)	5.7	269.4	
		~s(plant material)	4.7	**267.7**	0.0
Copepoda	Day-10	~Treatment + s(plant material, by= Treatment)	6.4	170.2	
		~Treatment + s(plant material)	5.1	170.3	
		~s(plant material)	4.2	**168.3**	0.0
	Day-31	~Treatment + s(plant material, by= Treatment)	11.1	**159.6**	-9.7
		~Treatment + s(plant material)	7.7	168.4	
		~s(plant material)	6.6	169.4	
	Day-59	~Treatment + s(plant material, by= Treatment)	8.0	181.1	
		~Treatment + s(plant material)	4.0	181.1	
		~s(plant material)	3.0	**180.2**	0.0
	Day-89	~Treatment + s(plant material, by= Treatment)	5.0	157.5	
		~Treatment + s(plant material)	4.0	155.6	
		~s(plant material)	3.0	**153.6**	0.0
Culex spp.	Day-10	~Treatment + s(plant material, by= Treatment)	8.7	**116.8**	-57.7
		~Treatment + s(plant material)	6.0	173.8	
		~s(plant material)	4.9	174.5	
	Day-31	~Treatment + s(plant material, by= Treatment)	10.9	**172.9**	-18.3
		~Treatment + s(plant material)	4.6	190.5	
		~s(plant material)	3.5	191.2	
	Day-59	~Treatment + s(plant material, by= Treatment)	17.8	**53.2**	-99.2
		~Treatment + s(plant material)	5.8	152.9	
		~s(plant material)	4.8	152.5	
	Day-89	~Treatment + s(plant material, by= Treatment)	5.0	**86.4**	-5.3
		~Treatment + s(plant material)	4.0	92.3	
		~s(plant material)	3.0	91.6	

**Treatment + s(plant material, by= Treatment)* GAM has parametric term (*Treatment*) and separate smoothers for each treatment. *Treatment + s(plant material)* GAM has a global smoother allowing for off-set intercepts according to treatments. The *s(plant material)* GAM fits a global smoother to all data. *Bold* represents the selected models. Delta AIC (*ΔAIC*) is the difference between the selected model and the global smoother model.

Table S8. Generalized additive models (GAM) testing fire treatment (burned vs. unburned) and factor-smooth interaction effects on zooplankton (Daphniidae, Copepoda) and mosquito larvae (*Culex* spp.) densities at four time points. Separate smoothers were fit for burned and unburned data, and anova tables were generated by *anova.gam*().

Taxa	*Time*	*Effect*	*df /edf*	*Ref.df*	*F*	*p-value*
Daphniidae						
	Day-10	s(plant material)	1.000	1.000	6.395	**0.017**
	Day-31	s(plant material)	2.849	3.492	2.998	**0.047**
	Day-59	Treatment	1.000	–	11.540	**0.002**
		s(plant material) : burned	2.296	2.826	1.482	0.194
		s(plant material) : unburned	1.000	1.000	0.470	0.499
	Day-89	s(plant material)	2.227	2.744	2.301	0.127
Copepoda						
	Day-10	s(plant material)	1.750	2.165	6.656	**0.004**
	Day-31	Treatment	1.000	–	3.968	0.068
		s(plant material) : burned	1.603	1.977	0.521	0.629
		s(plant material) : unburned	5.106	6.109	6.173	**0.001**
	Day-59	s(plant material)	1.000	1.000	2.496	0.125
	Day-89	s(plant material)	1.000	1.000	1.625	0.213
Culex spp.						
	Day-10	Treatment	1.000	–	17.600	**<0.001**
		s(plant material) : burned	1.000	1.000	2.611	0.120
		s(plant material) : unburned	4.436	5.357	62.444	**<0.001**
	Day-31	Treatment	1.000	–	5.655	**0.027**
		s(plant material) : burned	3.970	4.819	4.980	**0.003**
		s(plant material) : unburned	2.487	3.057	27.250	**<0.001**
	Day-59	Treatment	1.000	–	47.330	**<0.001**
		s(plant material) : burned	7.942	8.676	31.600	**<0.001**
		s(plant material) : unburned	6.417	7.470	188.400	**<0.001**
	Day-89	Treatment	1.000	–	1.588	0.219
		s(plant material) : burned	1.000	1.000	1.848	0.186
		s(plant material) : unburned	1.000	1.000	28.289	**<0.001**

Treatment indicates the parametric term in GAM, *s(plant material)* is the smooth term for either burned or unburned treatments. *df / edf* column indicates either *df* (degrees of freedom) for parametric terms or *edf* (effective degrees of freedom) for smoother terms; *Ref.df* = reference degree of freedom, where dashes indicate NA for parametric terms. Significant effects (p<0.05) are in bold.

Table S9. Model selection for flow cytometry-determined cell abundance for autofluorescent cells and total microbial cells (measured by Syber Green) with candidate GAM models* assessed at three time points post addition of plant material to experimental mesocosms.

Metric	*Time*	*Model*	*df*	*AIC*	*ΔAIC*
Autofluorescent cells	Day-10	~Treatment + s(plant material, by= Treatment)	5.0	910.4	
		~Treatment + s(plant material)	4.0	908.6	
		~s(plant material)	3.0	**907.0**	0.0
	Day-59	~Treatment + s(plant material, by= Treatment)	5.8	818.9	
		~Treatment + s(plant material)	4.9	818.3	
		~s(plant material)	3.6	**818.2**	0.0
	Day-89	~Treatment + s(plant material, by= Treatment)	7.5	**961.2**	-0.2
		~Treatment + s(plant material)	4.0	962.0	
		~s(plant material)	3.0	961.4	
Total microbial cells	Day-10	~Treatment + s(plant material, by= Treatment)	10.2	1017.6	
		~Treatment + s(plant material)	7.5	1011.3	
		~s(plant material)	6.5	**1010.2**	0.0
	Day-59	~Treatment + s(plant material, by= Treatment)	5.1	926.5	
		~Treatment + s(plant material)	4.0	925.5	
		~s(plant material)	3.0	**923.8**	0.0
	Day-89	~Treatment + s(plant material, by= Treatment)	5.0	**979.1**	-19.0
		~Treatment + s(plant material)	4.0	998.9	
		~s(plant material)	3.0	998.1	

**Treatment + s(plant material, by= Treatment)* GAM has parametric terms (*Treatment*) and separate smoothers for each treatment. *Treatment + s(plant material)* GAM has a global smoother allowing for off-set intercepts according to treatments. The *s(plant material)* GAM fits a global smoother to all data. Bold represents the selected models. Delta AIC (*ΔAIC*) is the difference between the selected model and the global smoother model.

Table S10. Generalized additive models (GAM) testing fire treatment (burned vs. unburned) and factor-smooth interaction effects on the abundance of autofluorescent and total microbial at three time points. Separate smoothers were fit for burned and unburned data, and anova tables were generated by *anova.gam*().

Metric-and-Time	*Effect*	*df /edf*	*Ref.df*	*F*	*p-value*
Autofluorescent cells					
Day-10	s(plant material)	1.000	1.001	0.018	0.894
Day-59	s(plant material)	1.363	1.635	0.207	0.694
Day-89	Treatment	1.000	–	1.522	0.228
	s(plant material) : burned	1.477	1.801	1.075	0.263
	s(plant material) : unburned	2.214	2.728	1.436	0.340
Total microbial cells					
Day-10	s(plant material)	3.844	4.673	4.304	**0.007**
Day-59	s(plant material)	1.001	1.001	0.006	0.945
Day-89	Treatment	1.000	–	2.177	0.151
	s(plant material) : burned	1.000	1.001	0.170	0.684
	s(plant material) : unburned	1.000	1.000	46.790	**<0.001**

Treatment indicates the parametric term in GAM, *s(plant material)* is the smooth term for either burned or unburned treatments. *df / edf* column indicates either *df* (degrees of freedom) for parametric terms or *edf* (effective degrees of freedom) for smoother terms; *Ref.df* = reference degree of freedom, where dashes indicate NA for parametric terms. Significant effects (p<0.05) are in bold.

Table S11. Model selection for carbon isotope values of carbon dioxide ($\delta^{13}CO_2$) and methane ($\delta^{13}CH_4$) with candidate GAM models* assessed at three time points post addition of plant material to experimental mesocosms.

Metric	*Time*	*Model*	*df*	*AIC*	*ΔAIC*
$\delta^{13}CO_2$	Day-0	~Treatment + s(plant material, by= Treatment)	5	128.0	
		~Treatment + s(plant material)	4	**126.4**	-3.8
		~s(plant material)	3	130.2	
	Day-10	~Treatment + s(plant material, by= Treatment)	8.7	135.9	
		~Treatment + s(plant material)	6.5	130.6	
		~s(plant material)	5.5	**129.1**	0
	Day-31	~Treatment + s(plant material, by= Treatment)	7	153.6	
		~Treatment + s(plant material)	5.6	149.8	
		~s(plant material)	4.6	**148.9**	0
	Day-59	~Treatment + s(plant material, by= Treatment)	9	**169.3**	-1.8
		~Treatment + s(plant material)	4	170.7	
		~s(plant material)	3	171.0	
$\delta^{13}CH_4$	Day-0	~Treatment + s(plant material, by= Treatment)	6.4	30.2	
		~Treatment + s(plant material)	5.3	29.0	
		~s(plant material)	4.4	**27.1**	0.0
	Day-10	~Treatment + s(plant material, by= Treatment)	7.6	13.7	
		~Treatment + s(plant material)	6.2	12.8	
		~s(plant material)	5.2	**11.7**	0.0
	Day-31	~Treatment + s(plant material, by= Treatment)	7.4	20.1	
		~Treatment + s(plant material)	6.8	13.7	
		~s(plant material)	5.8	**12.8**	0.0
	Day-59	~Treatment + s(plant material, by= Treatment)	8.4	**69.0**	-4.8
		~Treatment + s(plant material)	6.9	75.6	
		~s(plant material)	6.0	73.8	

**Treatment + s(plant material, by= Treatment)* GAM has parametric terms (*Treatment*) and separate smoothers for each treatment. *Treatment + s(plant material)* GAM has a global smoother allowing for off-set intercepts according to treatments. The *s(plant material)* GAM fits a global smoother to all data. Bold represents the selected models. Delta AIC (*ΔAIC*) is the difference between the selected model and the global smoother model.

Table S12. Generalized additive models (GAM) testing fire treatment (burned vs. unburned) and factor-smooth interaction effects on the carbon isotope values of carbon dioxide ($\delta^{13}CO_2$) and methane ($\delta^{13}CH_4$) at three time points. Separate smoothers were fit for burned and unburned data, and anova tables were generated by *anova.gam*().

Metric	*Time*	*Effect*	*df /edf*	*Ref.df*	*F*	*p-value*
$\delta^{13}CO_2$						
	Day-0	Treatment	1.000	–	5.711	**0.024**
		s(plant material)	1.000	1.000	0.611	0.441
	Day-10	s(plant material)	2.904	3.559	13.320	**<0.001**
	Day-59	s(plant material)	2.104	2.596	19.000	**<0.001**
	Day-89	Treatment	1.000	–	2.703	0.114
		s(plant material) : burned	3.051	3.735	4.359	**0.014**
		s(plant material) : unburned	1.793	2.219	7.092	**0.003**
$\delta^{13}CH_4$						
	Day-0	s(plant material)	1.928	2.384	1.275	0.376
	Day-10	s(plant material)	2.589	3.180	4.048	**0.017**
	Day-59	s(plant material)	3.093	3.785	2.746	0.060
	Day-89	Treatment	1.000	–	0.200	0.659
		s(plant material) : burned	3.879	4.714	3.399	**0.021**
		s(plant material) : unburned	1.000	1.000	3.595	0.071

Treatment indicates the parametric term in GAM, *s(plant material)* is the smooth term for either burned or unburned treatments. *df / edf* column indicates either *df* (degrees of freedom) for parametric terms or *edf* (effective degrees of freedom) for smoother terms; *Ref.df* = reference degree of freedom, where dashes indicate NA for parametric terms. Significant effects ($p<0.05$) are in bold.

Table S13. Model selection for microbial genome characteristics and alpha diversity with candidate GAM models* assessed at three time points post addition of plant material to experimental mesocosms.

Metric	*Time*	*Model*	*df*	*AIC*	*ΔAIC*
Number of coding sequences					
	Day-10	~Treatment + s(plant material, by= Treatment)	5.8	432.0	
		~Treatment + s(plant material)	5.8	**428.7**	-2.0
		~s(plant material)	4.5	430.7	
	Day-59	~Treatment + s(plant material, by= Treatment)	5.5	423.5	
		~Treatment + s(plant material)	4.0	421.5	
		~s(plant material)	3.0	**419.6**	0.0
	Day-89	~Treatment + s(plant material, by= Treatment)	6.6	445.2	
		~Treatment + s(plant material)	5.0	445.8	
		~s(plant material)	4.1	**444.7**	0.0
Genomic plasticity (phi)					
	Day-10	~Treatment + s(plant material, by= Treatment)	8.5	-108.5	
		~Treatment + s(plant material)	6.3	-112.4	
		~s(plant material)	5.3	**-114.4**	0.0
	Day-59	~Treatment + s(plant material, by= Treatment)	5.0	-111.2	
		~Treatment + s(plant material)	4.0	-111.5	
		~s(plant material)	3.0	**-112.8**	0.0
	Day-89	~Treatment + s(plant material, by= Treatment)	5.0	-107.4	
		~Treatment + s(plant material)	4.0	-109.1	
		~s(plant material)	3.0	**-110.8**	0.0
Genome size (bp)					
	Day-10	~Treatment + s(plant material, by= Treatment)	6.8	858.9	
		~Treatment + s(plant material)	6.2	**853.8**	-2.0
		~s(plant material)	5.1	855.8	
	Day-59	~Treatment + s(plant material, by= Treatment)	5.0	840.4	
		~Treatment + s(plant material)	4.0	838.9	
		~s(plant material)	3.0	837.1	0.0
	Day-89	~Treatment + s(plant material, by= Treatment)	6.9	**875.7**	-0.8
		~Treatment + s(plant material)	5.0	877.2	
		~s(plant material)	4.0	876.5	
Doubling time (gRodon)					
	Day-10	~Treatment + s(plant material, by= Treatment)	9.6	**66.6**	-1.6
		~Treatment + s(plant material)	6.3	68.5	
		~s(plant material)	5.3	68.2	
	Day-59	~Treatment + s(plant material, by= Treatment)	6.3	60.1	
		~Treatment + s(plant material)	5.3	57.2	
		~s(plant material)	4.3	**55.4**	0.0
	Day-89	~Treatment + s(plant material, by= Treatment)	5.0	82.6	
		~Treatment + s(plant material)	4.2	81.2	
		~s(plant material)	3.3	**79.3**	0.0

Table S13. (continued)

Microbial alpha diversity (Shannon)					
	Day-10	~Treatment + s(plant material, by= Treatment)	9.5	4.4	
		~Treatment + s(plant material)	6.1	**3.5**	-2.1
		~s(plant material)	5.1	5.6	
	Day-59	~Treatment + s(plant material, by= Treatment)	8.4	**21.6**	-4.4
		~Treatment + s(plant material)	6.1	27.8	
		~s(plant material)	5.2	26.0	
	Day-89	~Treatment + s(plant material, by= Treatment)	11.3	**36.5**	-10.2
		~Treatment + s(plant material)	6.0	48.3	
		~s(plant material)	5.1	46.7	

**Treatment + s(plant material, by= Treatment)* GAM has parametric terms (*Treatment*) and separate smoothers for each treatment. *Treatment + s(plant material)* GAM has a global smoother allowing for off-set intercepts according to treatments. The *s(plant material)* GAM fits a global smoother to all data. Bold represents the selected models. Delta AIC (*ΔAIC*) is the difference between the selected model and the global smoother model.

Table S14. Generalized additive models (GAMs) testing fire treatment (burned vs. unburned) and factor-smooth interaction effects on inferred microbial genome characteristics and alpha diversity at three time points. Separate smoothers were fit for burned and unburned data, and anova tables were generated by *anova.gam*().

Metric	*Time*	*Effect*	*df /edf*	*Ref.df*	*F*	*p-value*
Number of coding sequences						
	Day-10	Treatment	1.000	–	3.609	**0.069**
		s(plant material)	2.276	2.802	4.723	**0.014**
	Day-59	s(plant material)	1.001	1.001	0.477	0.496
	Day-89	s(plant material)	1.664	2.057	0.740	0.491
Genomic plasticity (phi)						
	Day-10	s(plant material)	2.754	3.379	22.430	**<0.001**
	Day-59	s(plant material)	1.000	1.000	14.370	**<0.001**
	Day-89	s(plant material)	1.000	1.001	0.162	0.690
Genome size (bp)						
	Day-10	Treatment	1.000	–	3.531	0.072
		s(plant material)	2.625	3.224	7.653	**<0.001**
	Day-59	s(plant material)	1.001	1.001	0.056	0.815
	Day-89	Treatment	1.000	–	1.246	0.275
		s(plant material) : burned	2.379	2.927	1.514	0.208
		s(plant material) : unburned	1.000	1.001	0.241	0.628
Doubling time (gRodon)						
	Day-10	Treatment	1.000	–	1.759	0.198
		s(plant material) : burned	2.554	3.138	3.112	**0.045**
		s(plant material) : unburned	2.856	3.501	1.831	0.128
	Day-59	s(plant material)	1.870	2.301	7.831	**0.002**
	Day-89	s(plant material)	1.172	1.322	0.624	0.578
Microbial alpha diversity (Shannon)						
	Day-10	Treatment	1.000	–	3.749	0.064
		s(plant material)	2.748	2.372	6.714	**0.001**
	Day-59	Treatment	1.000	–	0.394	0.536
		s(plant material) : burned	3.090	3.706	4.758	**0.008**
		s(plant material) : unburned	1.422	1.724	0.508	0.479
	Day-89	Treatment	1.000	–	0.662	0.425
		s(plant material) : burned	3.435	4.190	6.196	**0.002**
		s(plant material) : unburned	3.401	4.151	1.452	0.259

Treatment indicates the parametric term in GAM, *s(plant material)* is the smooth term for either burned or unburned treatments. *df / edf* column indicates either *df* (degrees of freedom) for parametric terms or *edf* (effective degrees of freedom) for smoother terms; *Ref.df* = reference degree of freedom, where dashes indicate NA for parametric terms. Significant effects ($p<0.05$) are in bold.

Table S15. PERMANOVA results for effects of experimental conditions (burning treatment, mass of plant material added, and time) on composition of the zooplankton community (relative abundances).

Source of Variation	*df*	*SS*	R^2	*F*	*p-value*
Burning Treatment	1	0.823	0.023	4.869	**0.004**
Plant Material	1	3.632	0.103	21.486	**0.001**
Time	1	8.805	0.249	13.023	**0.001**
Residual	131	22.143	0.625		
Total	137	35.402	1.000		

PERMANOVA table generated from 999 permutations and Bray-Curtis dissimilarity. *df*=degrees of freedom; *SS*=sum of squares. Significant effects ($p<0.05$) are in bold.

Table S16. PERMANOVA results for effects of experimental conditions (burning treatment, mass of plant material added, and time) on microbial metabolic pathways counts for water-associated microbes. Microbial metabolic pathways were identified with paprica.

Source of Variation	*df*	*SS*	R^2	*F*	*p-value*
Burning Treatment	1	0.042	0.012	1.126	0.301
Plant Material	1	0.103	0.029	2.751	**0.044**
Time	1	0.244	0.069	3.267	**0.006**
Residual	84	3.134	0.890		
Total	88	3.523	1.000		

PERMANOVA table generated from 999 permutations and Bray-Curtis dissimilarity (based on raw metabolic pathway counts). *df*=degrees of freedom; *SS*=sum of squares. Significant effects ($p<0.05$) are in bold.

Supplemental Figures

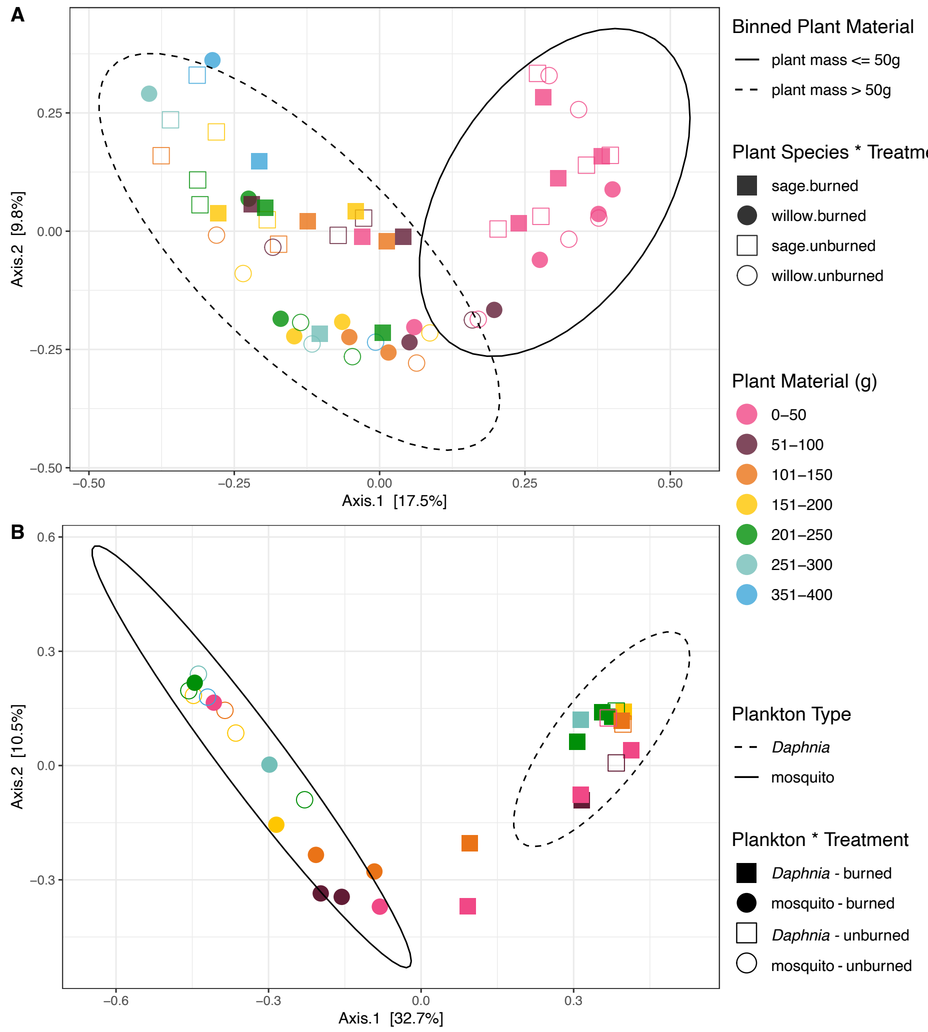

Figure S1. Principle coordinate analysis (PCoA) showing variation in (**A**) plant-associated microbiome composition and (**B**) zooplankton-associated microbiome composition 3 months after plant material was added. Color indicates binned amount of plant material added, and shape represents treatment and plant species or zooplankton taxa. Ellipses represent the most significant predictor of microbiome variation (**Table S5, S6**); (**A**) plant mass (specifically at the 50g threshold) explains 11.6% of plant microbiome variation, and (**B**) zooplankton type explains 26.9% of zooplankton microbiome variation.

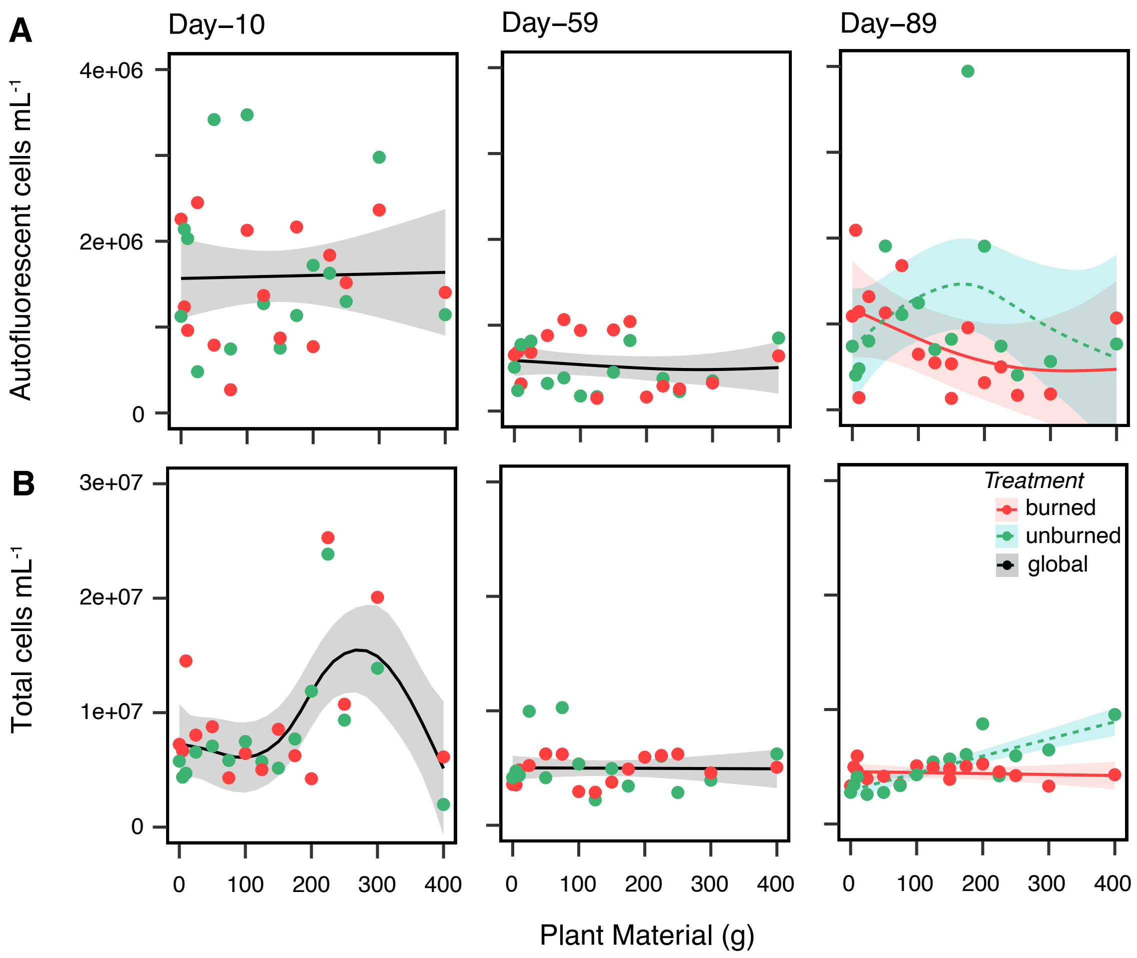

Figure S2. Flow cytometry (cells mL^{-1}) measurements of (**A**) autofluorescence and (**B**) total microbial cells in experimental mesocosms 10, 59, and 89 days after receiving differing amounts of burned or unburned plant material. Lines represent best-fit generalized additive models (GAMs) with 95% confidence intervals. Black lines with gray confidence intervals indicate global smoothers across all data points; colored lines indicate factor-smooths that vary between treatments. GAM model fits can be found in **Tables S9, S10**, and model treatment differences for Day-89 where treatment-specific smoothers were fit can be found in **Fig S3**.

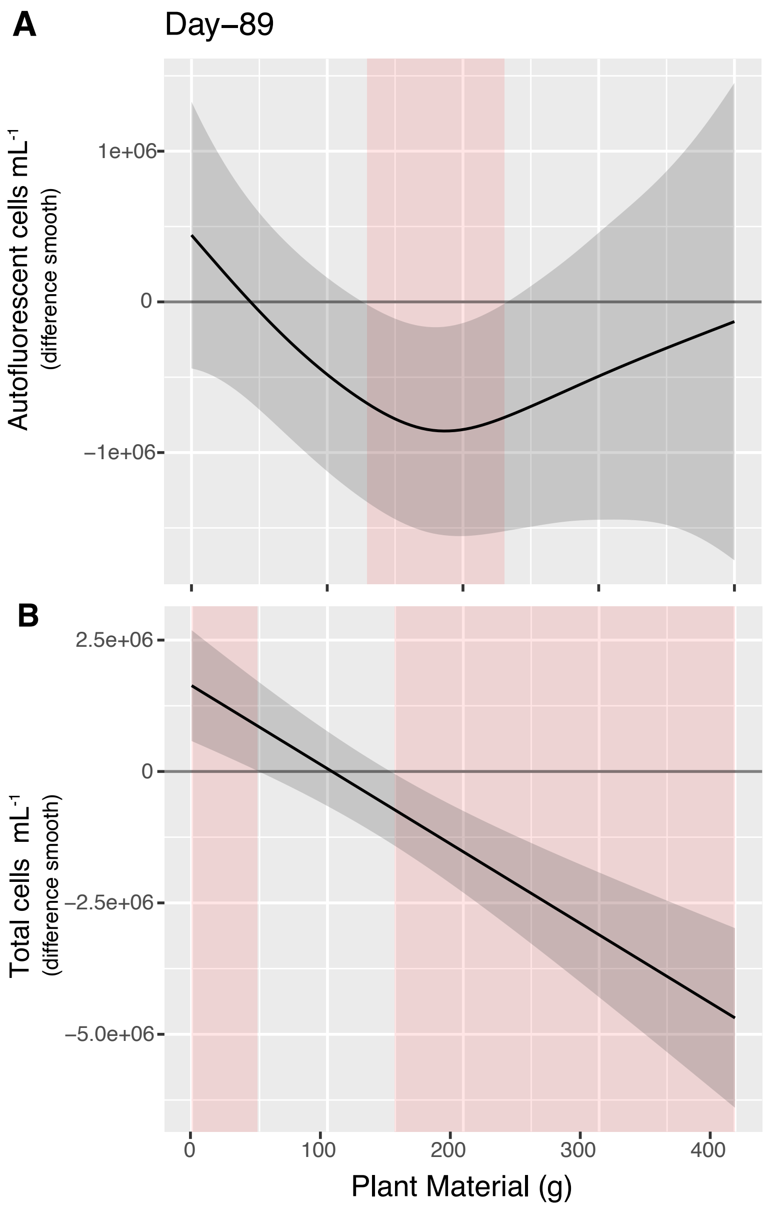

Figure S3. Model effects from GAMs with differences between smoothers for (**A**) autofluorescent and (**B**) total microbial cells 89-days after receiving burned and unburned plant material. Shaded regions are the confidence interval for 'the difference smooth,' which is the difference between burned and unburned treatment smoothers. Significant differences between treatment-level smoothers are noted in regions that do not include zero ± model confidence intervals and are shaded in pink.

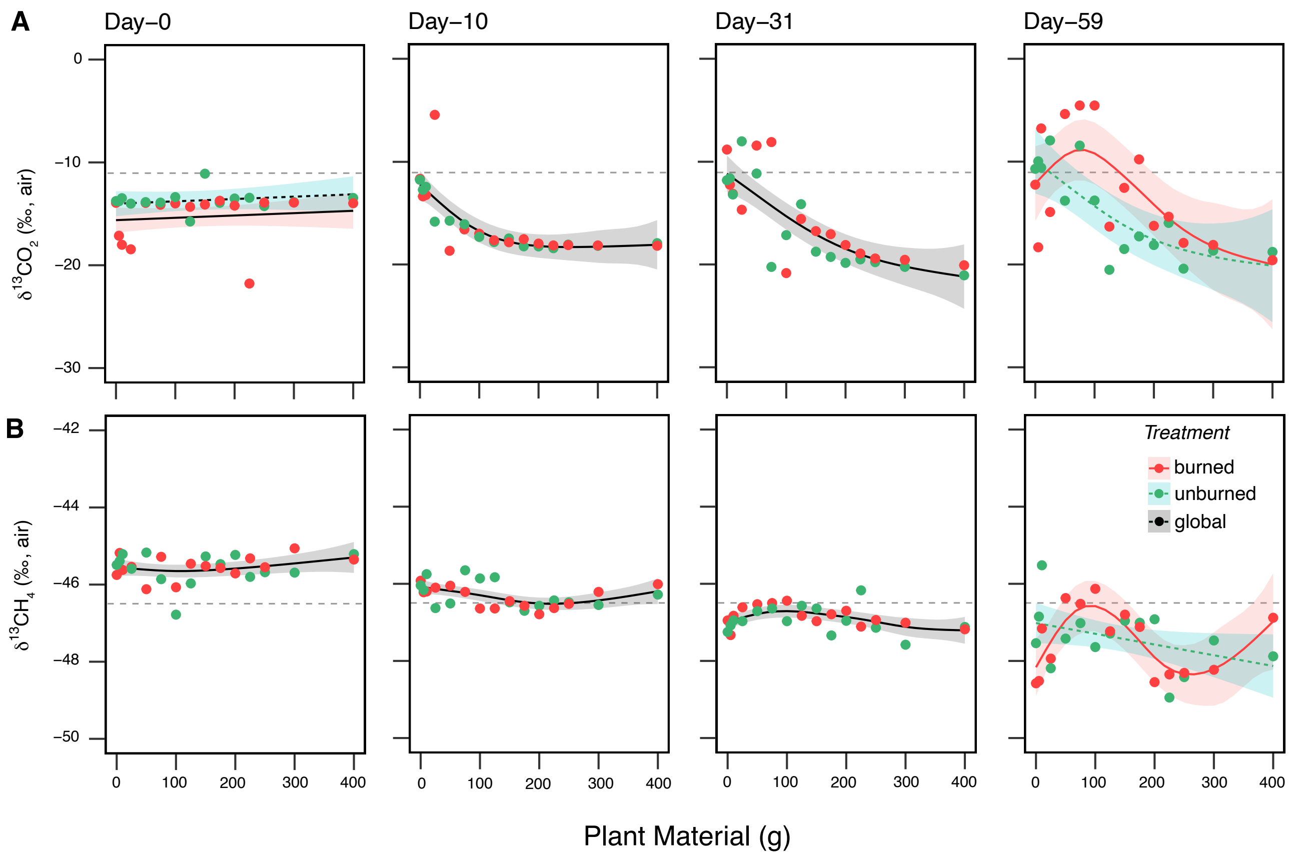

Figure S4. Stable isotope analysis of (**A**) $\delta^{13}CO_2$ and (**B**) $\delta^{13}CH_4$ in mesocosms receiving burned and unburned plant material at the beginning of the experiment and during three experimental time points. Horizontal dashed lines (*gray*) indicate atmospheric isotope values for carbon dioxide or methane at each sampling day. Lines represent best-fit generalized additive models (GAMs) with 95% confidence intervals. Black lines with gray confidence intervals indicate global smoothers across all data points; solid (*burned*) and dotted (*unburned*) black lines together represent treatment-level intercepts with global smoothers; colored lines indicate factor-smooths that vary between treatments. GAM model fits can be found in **Tables S11, S12**, and model treatment differences for Day-59 where treatment-specific smoothers were fit can be found in **Fig S5.**

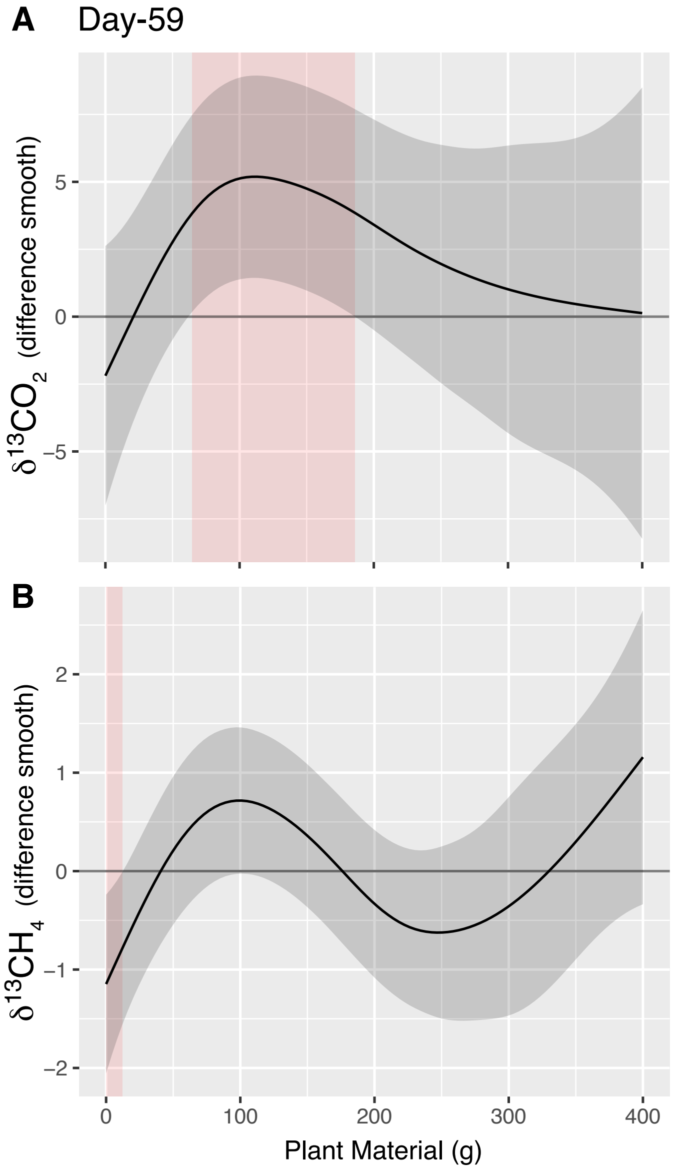

Figure S5. Model effects from GAMs with differences between smoothers for (**A**) $\delta^{13}CO_2$ and (**B**) $\delta^{13}CH_4$ 59-days after experimental mesocosms received burned or unburned plant material. Shaded regions are the confidence interval for 'the difference smooth,' which is the difference between burned and unburned treatment smoothers. Significant differences between treatment-level smoothers are noted in regions that do not include zero ± model confidence intervals and are shaded in pink.

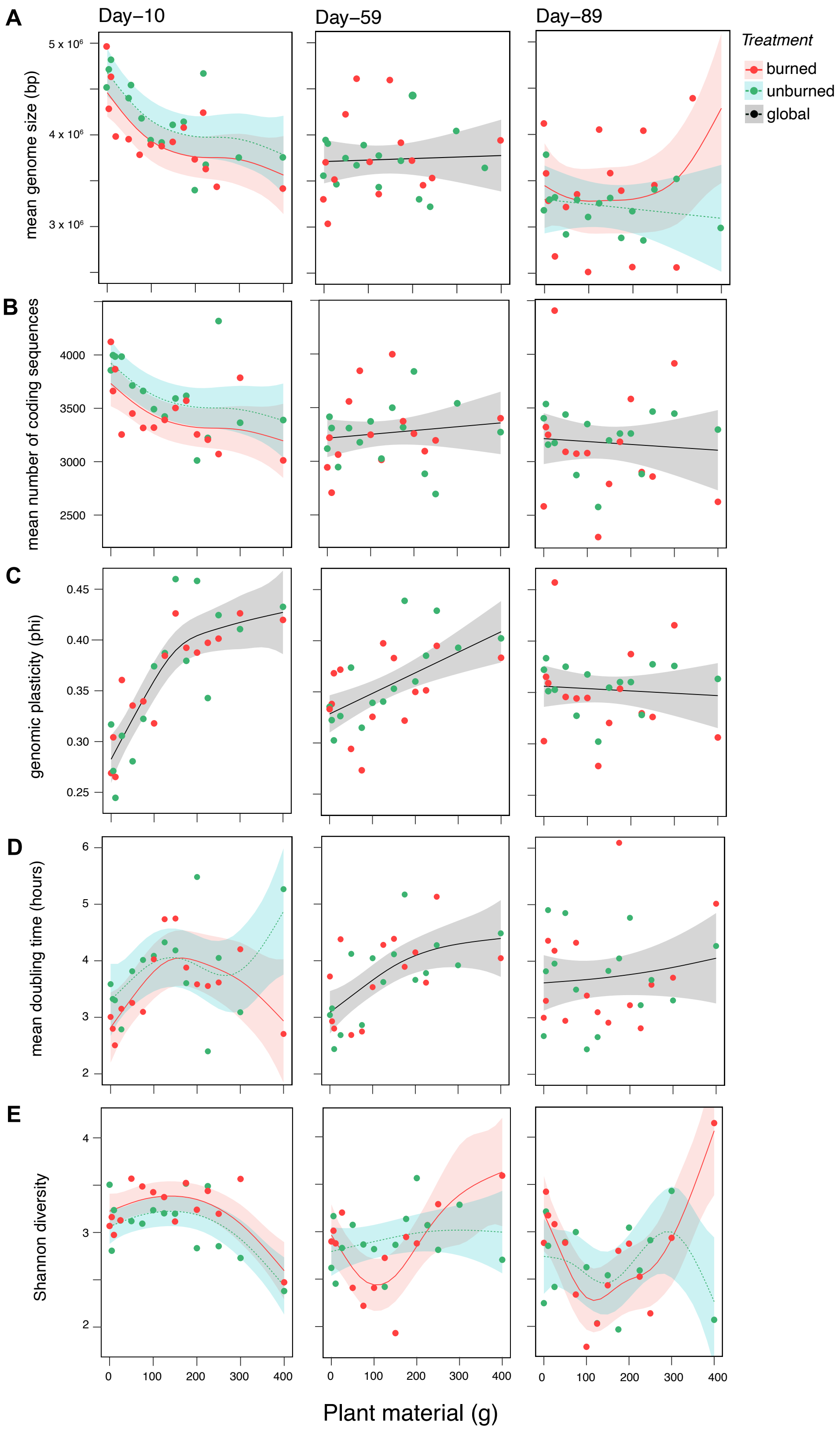

A
B
C
D
E
Day−10
Day−59
Day−89
Treatment
burned
unburned
global
mean genome size (bp)
5 x 10⁶
4 x 10⁶
3 x 10⁶
mean number of coding sequences
4000
3500
3000
2500
genomic plasticity (phi)
0.45
0.40
0.35
0.30
0.25
mean doubling time (hours)
6
5
4
3
2
Shannon diversity
4
3
2
0
100
200
300
400
Plant material (g)

Figure S6. Variation across time and mesocosms for (**A**) mean number of coding sequences; (**B**) mean genome size (bp); (**C**) relative mean genomic plasticity (phi), a comparison of microbe-specific genomes to the mean genome for a clade; (**D**) mean estimated doubling time (hours); (**E**) mean microbial alpha diversity, quantified using the Shannon diversity metric. Lines represent best-fit generalized additive models (GAMs) with 95% confidence intervals. Black lines with gray confidence intervals indicate global smoothers across all data points; solid (*burned*) and dotted (*unburned*) black lines together represent treatment-level intercepts with global smoothers; colored lines indicate factor-smooths that vary between treatments. GAM model fits can be found in **Tables S13, S14**, and model treatment differences where treatment-specific smoothers were fit can be found in **Fig S7.**

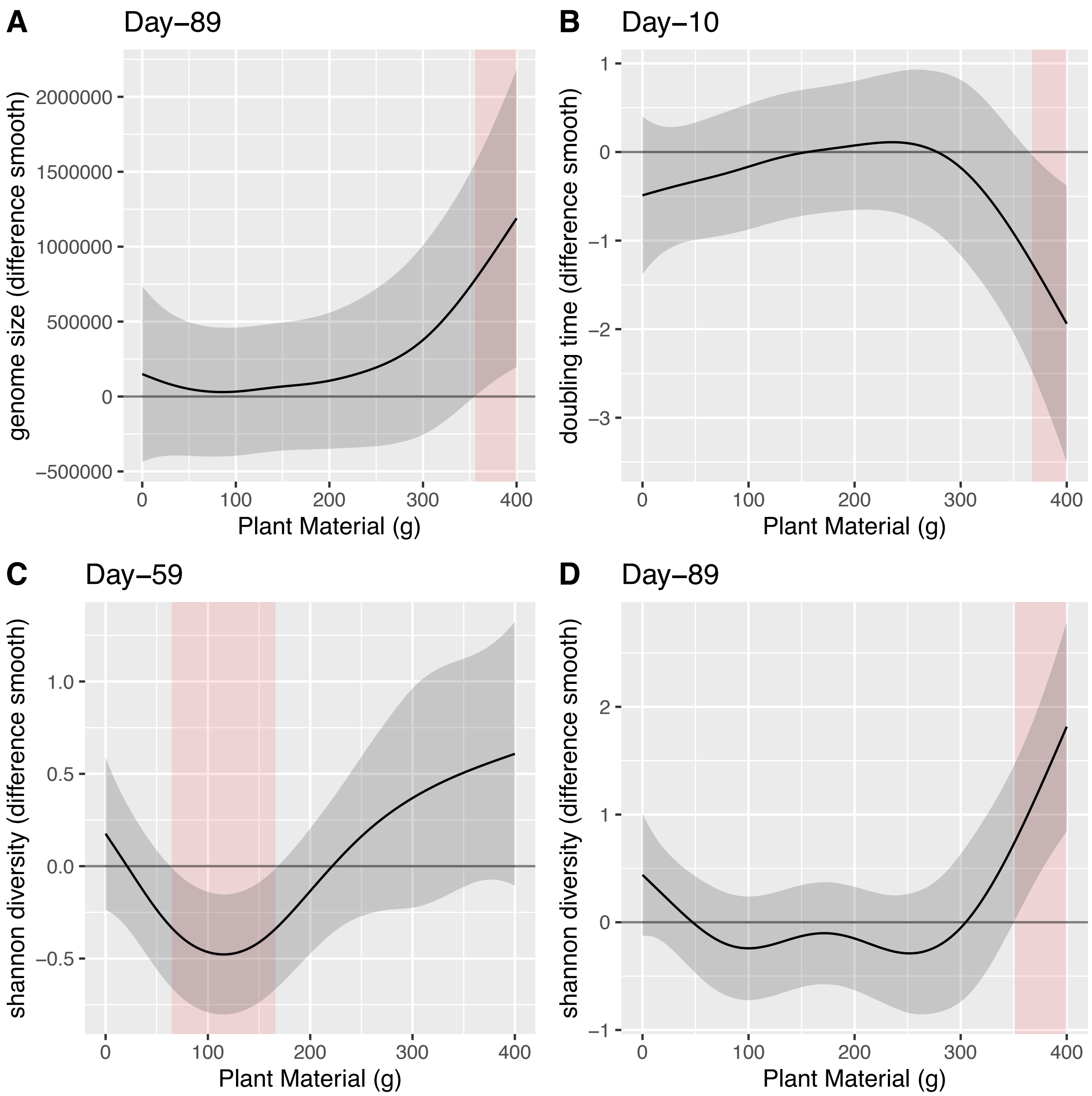

Figure S7. Model effects from GAMs with differences between smoothers for (**A**) bacterial genome size on Day-89; (**B**) bacterial doubling time on Day-10; (**C**) bacterial Shannon diversity on Day-59; and (**D**) bacterial Shannon diversity on Day-89. Shaded regions are the confidence interval for ‘the difference smooth,’ which is the difference between burned and unburned treatment smoothers. Significant differences between treatment-level smoothers are noted in regions that do not include zero ± model confidence intervals and are shaded in pink.

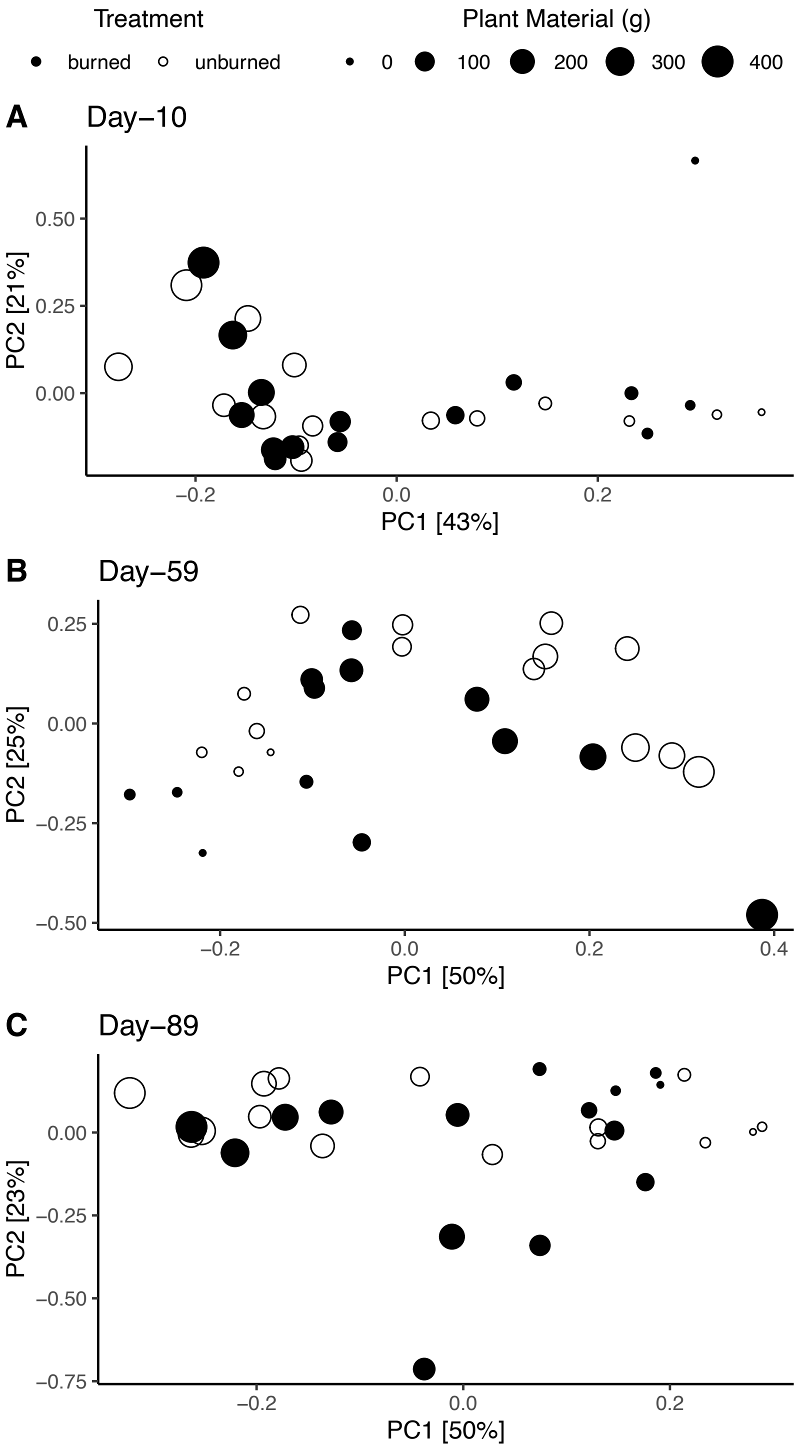

Figure S8. PCoAs of environmental variables (temperature, pH, %DO, [DOC], FI, BIX, HIX, Sr, and $SUVA_{254}$) on (**A**) Day-10, (**B**) Day-59, and (**C**) Day-89. Point shape (filled or open circles) represents burning-treatment, and size represents amount of plant material added.

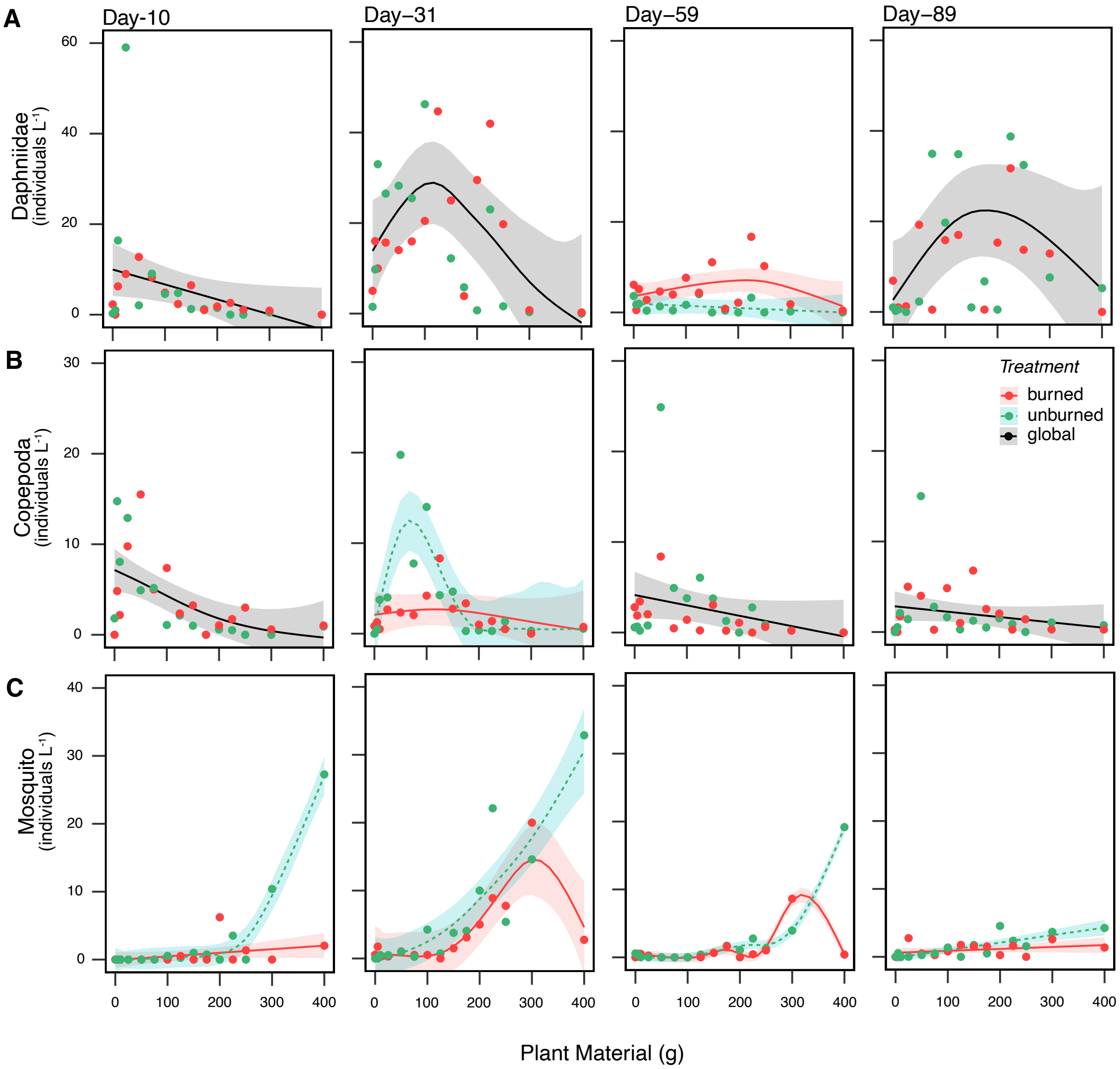

Figure S9. Zooplankton densities for members of (**A**) family Daphniidae (*Daphnia*, *Ceriodaphnia*, *Bosmina*), (**B**) class Copepoda (calanoids, cyclopoids, nauplii), and (**C**) mosquito larvae (*Culex* spp.) in burned and unburned experimental mesocosms on Days-10, 31, 59, and 89. Lines represent best-fit generalized additive models (GAMs) with 95% confidence intervals. Black lines with gray confidence intervals indicate global smoothers across all data points; solid (*burned*) and dotted (*unburned*) black lines together represent treatment-level intercepts with

global smoothers; colored lines indicate factor-smooths that vary between treatments. GAM model fits can be found in **Tables S7, S8**, and model treatment differences for Day-59 where treatment-specific smoothers were fit can be found in **Fig S10.**

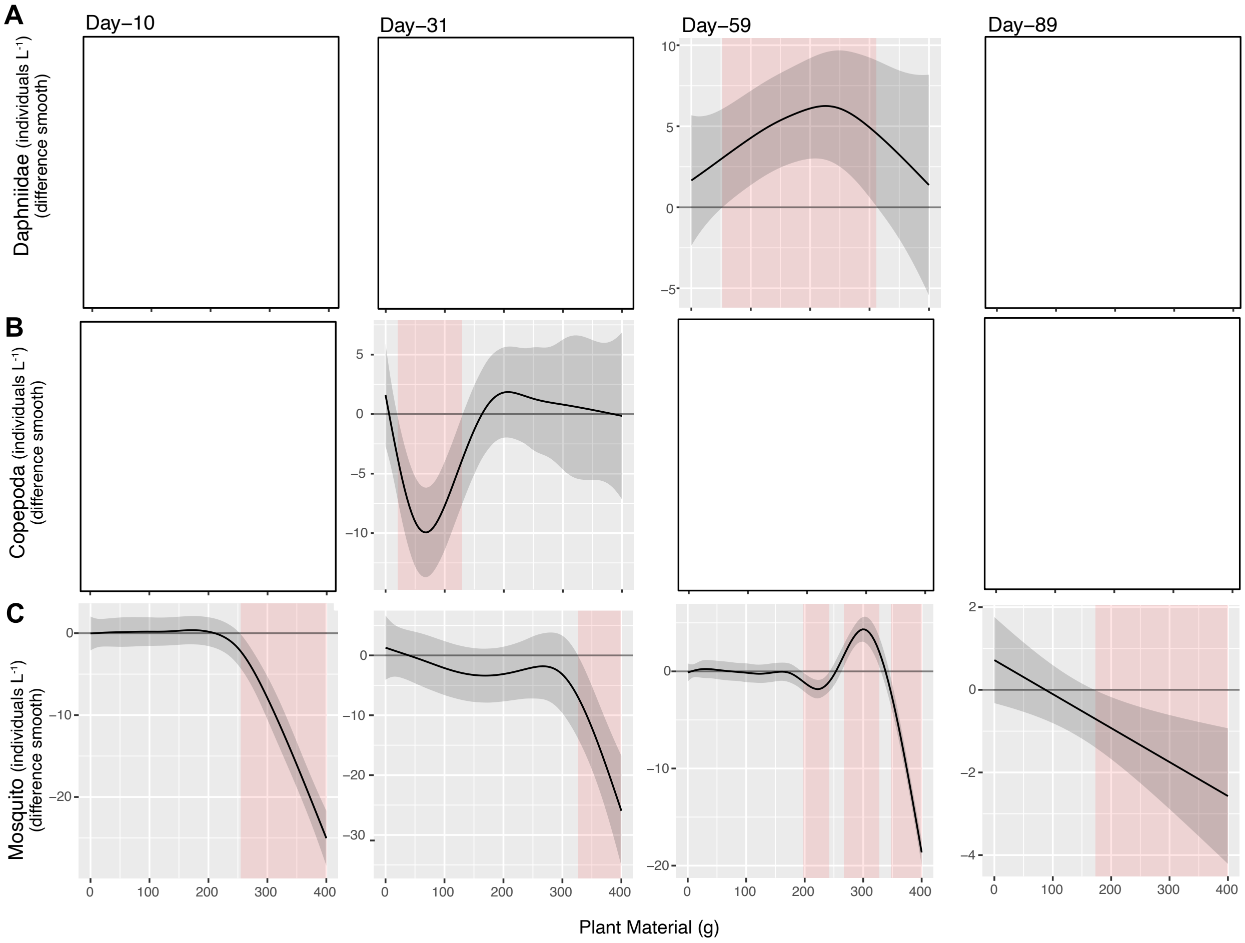

Figure S10. Model effects from GAMs with differences between smoothers for (**A**) family Daphniidae (*Daphnia*, *Ceriodaphnia*, *Bosmina*), (**B**) class Copepoda (calanoids, cyclopoids, nauplii), and (**C**) mosquito larvae (*Culex* spp.) Shaded regions are the confidence interval for 'the difference smooth,' which is the difference between burned and unburned treatment smoothers. Significant differences between treatment-level smoothers are noted in regions that do not include zero ± model confidence intervals and are shaded in pink. Blank plots represent non-significant differences between smoothers.

Supplemental References

1. Walters W, Hyde ER, Berg-Lyons D, Ackermann G, Humphrey G, Parada A, et al. Improved Bacterial 16S rRNA Gene (V4 and V4-5) and Fungal Internal Transcribed Spacer Marker Gene Primers for Microbial Community Surveys. *mSystems* 2016; **1**.
2. Caporaso JG, Lauber CL, Walters WA, Berg-Lyons D, Huntley J, Fierer N, et al. Ultra-high-throughput microbial community analysis on the Illumina HiSeq and MiSeq platforms. *ISME J* 2012; **6**: 1621–1624.
3. Wilson JM, Erazo N, Connors E, Chamberlain EJ, Clements SM, Carter ML, et al. Substantial microbial community shifts in response to an exceptional harmful algal bloom in coastal Southern California. *Elementa (Wash, DC)* 2022; **10**.
4. Simpson GL. gratia: Graceful ’ggplot’-based graphics and other functions for GAMs fitted using “mgcv.” *R package version 073* 2022.
5. Coretta S, van Rij J, Wieling M. Tidymv: tidy model visualisation for generalised additive models. *R package version* 2022.
